# Synthesis and Self-Assembly of Chiral Cylindrical Molecular Complexes: Functional Heterogeneous Liquid-Solid Materials Formed by Helicene Oligomers

**DOI:** 10.3390/molecules23020277

**Published:** 2018-01-29

**Authors:** Nozomi Saito, Masahiko Yamaguchi

**Affiliations:** Department of Organic Chemistry, Graduate School of Pharmaceutical Sciences, Tohoku University, Sendai 980-8578, Japan; nsaito@m.tohoku.ac.jp

**Keywords:** cylindrical, double-helix, helicene oligomers, self-assembly, heterogeneous liquid-solid, molecular complex

## Abstract

Chiral cylindrical molecular complexes of homo- and hetero-double-helices derived from helicene oligomers self-assemble in solution, providing functional heterogeneous liquid-solid materials. Gels and liotropic liquid crystals are formed by fibril self-assembly in solution; molecular monolayers and fibril films are formed by self-assembly on solid surfaces; gels containing gold nanoparticles emit light; silica nanoparticles aggregate and adsorb double-helices. Notable dynamics appears during self-assembly, including multistep self-assembly, solid surface catalyzed double-helix formation, sigmoidal and stairwise kinetics, molecular recognition of nanoparticles, discontinuous self-assembly, materials clocking, chiral symmetry breaking and homogeneous-heterogeneous transitions. These phenomena are derived from strong intercomplex interactions of chiral cylindrical molecular complexes.

## 1. Heterogeneous Liquid-Solid Materials

Heterogeneous liquid-solid materials can be constructed by self-assembly of materials derived from molecules [1,2,3,4,5,6,7,8,9,10,11,12,13,14,15,16]. Self-assembly involves the noncovalent bond formation, which has several advantages compared with the covalent bond formation: (1) Various shapes and structures of self-assembly materials can be formed by tuning the molecular structures; (2) diverse mechanisms of noncovalent bond formation can be used, including hydrogen-bonding, electrostatic interactions, van der Waals interactions, solvophobic interactions, π-π interactions and dipole-dipole interactions; (3) various spatial interactions including point-to-point, point-to-fiber, fiber-to-fiber, fiber-to-face and face-to-face modes are available, which are not restricted to the point-to-point interactions, that characterize covalent bond formation; (4) properties of the interactions can be affected largely by conditions such as temperature, concentration, light and chemical substances; (5) self-assembly materials can be degraded without using much energy and the constituent molecules may be recovered and reused.

**Figure 1:**
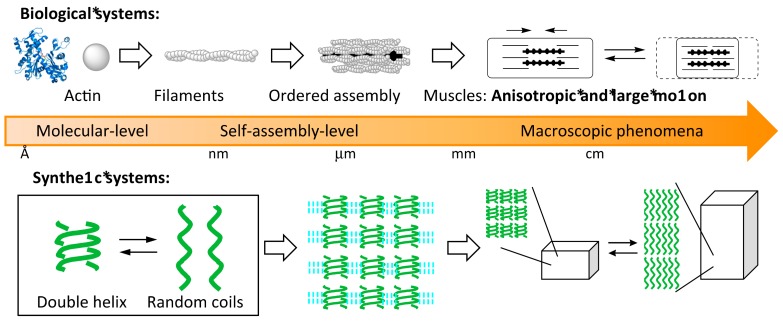
Bottom-up formation of heterogeneous liquid-solid systems derived from self-assembly of molecules. Both biological and synthetic systems are shown.

Structural switching in heterogeneous liquid-solid materials containing self-assembly materials is notable and is also essential for living things (Figure 1). For example, muscles move mechanically through heterogeneous liquid-solid materials involving myosin and actin proteins. Proteins and particles are transported on microtubules by kinesin proteins in water. Heterogeneous liquid-solid materials are important as media for chemical reactions, for example, as protein receptor bound on cell surfaces. These systems involve the interconversions among different ordered structures in self-assembly materials.

Another subject related to the dynamics of heterogeneous liquid-solid materials is the process of forming self-assembly materials from dispersed molecules in solution. Molecules interact to form nanometer-sized aggregates, which grow to form regular structures such as micrometer-sized fibrils and membranes in solution, which are heterogeneous liquid-solid materials. Mechanisms underlying this dynamic are important to understand and to control the structures and properties of heterogeneous liquid-solid materials, which, however, are often extremely complex. For example, nucleation-growth mechanisms have been examined to clarify such self-assembly, during which the initial formation of small nuclei is slow and the subsequent growth is fast, which provides sigmoidal kinetics [17,18,19,20,21,22].

**Figure 2:**
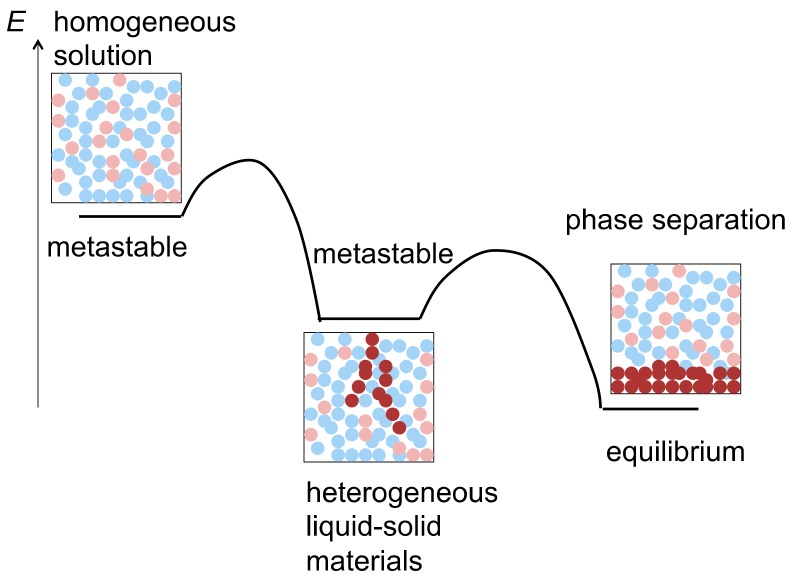
Energy aspects of homogeneous-heterogeneous transition in formation of heterogeneous liquid-solid materials involving self-assembly materials for solid phase.

Self-assembly phenomena involve changes of molecular dispersed homogeneous solutions into heterogeneous liquid-solid materials, being homogeneous-heterogeneous transitions, which seems to run counter to thermodynamics (Figure 2). Free energy is minimized and entropy maximized in a homogeneous solution, which is thermodynamically the most stable [23]. Such a homogeneous-heterogeneous transition does not occur in an equilibrium-to-equilibrium reaction, when the initial homogeneous solution is equilibrium [24,25,26,27]. On the other hand, a transition occurs, when the initial homogeneous solution is metastable and when such transition is an energetically downhill process from a metastable state to an equilibrium. Homogeneous metastable state can be generated by providing external energy such as heating, irradiating, or adding a chemical substance. Then, various intermediate states appear during the transition from a homogeneous metastable state to an equilibrium state in time-dependent manner.

Reversibility is also an interesting dynamic aspect of the homogeneous-heterogeneous transitions involving self-assembly. The original homogeneous state can be regenerated by providing suitable energy, such as heating or irradiation. Alternatively, addition and removal of chemical substances providing chemical energy can also be employed. When energy and matter are exchanged between the inside and outside of a system during a transition, the system is called open; when only energy is exchanged, the system is called closed. Such reversible systems of self-assembly materials exhibit interesting switching properties.

## 2. Self-Assembly of Chiral Cylindrical Molecular Complexes

### 2.1. Cylindrical Molecular Complexes

**Figure 3:**
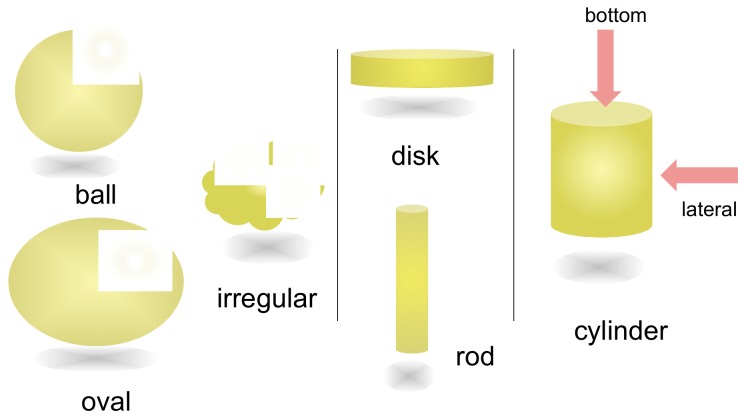
Various shapes of molecules and molecular complexes.

To construct functional heterogeneous liquid-solid materials involving self-assembly materials, the structures of molecules or molecular complexes, which are the structural units, are critical. Here, a molecular complex implies a small aggregate derived from molecules; for example, a double-helix. Principles to govern the structure and function of the self-assembly materials remain undeveloped and an approach to this subject is provided by the three-dimensional shape of molecules and molecular complexes (Figure 3). Rod- and disk-shaped molecules form self-assemblies such as membranes and micelles. Ball-, oval- and irregularly space shaped molecules, in contrast, are less common, which may be ascribed to fewer contact points for interactions with other molecules and to isotropy in shape. Among various shapes, cylindrical molecular complexes, being anisotropic in shape, are interesting, which can be regarded as a combination of a disk shape and a rod shape, both of which are anisotropic. The ideal cylindrical shape has *D*_∞*h*_ symmetry with broad surfaces on the lateral side and two bottoms. The lateral and bottom surfaces can be used to interact to form an anisotropic self-assembly material. As described by the Onsager theory [28,29], directional shapes and interactions between the shapes are important for constructing anisotropic systems such as liquid crystals.

**Figure 4:**
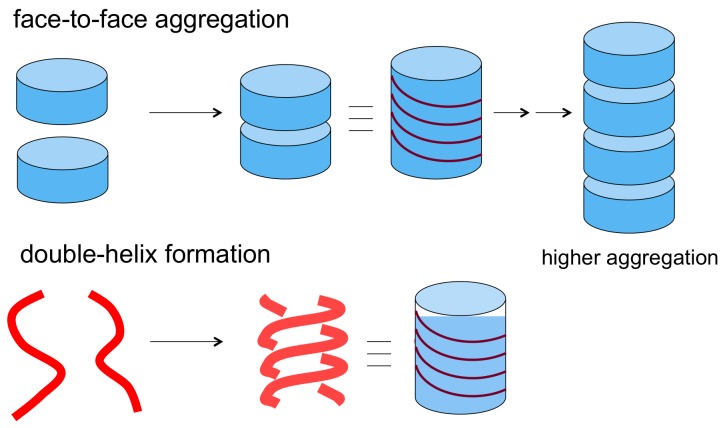
Methods of synthesis of cylindrical structures by molecular complexation.

Chiral cylindrical shapes of synthetic molecular complex are not common, which is in contrast to the case in biology, where many chiral cylindrical molecular complexes exist, as exemplified by double-stranded DNA and α-helix proteins [2,3,4,30,31]. The lack of synthetic chiral cylindrical molecular complex is ascribed to the lack of an appropriate synthetic method. Organic molecules with a cylindrical shape are not easy to synthesize employing covalent bond formation. However, such a shape can be relatively readily obtained using molecular complexes formed by noncovalent bond. One of conceivable methods is the use of disk-shaped molecules that aggregate at their bottom faces by face-to-face interactions, which have been observed for polycyclic aromatic molecules (Figure 4) [32,33]. Modification of aromatic structures at peripheral sites can be conducted, which has, however, a relatively limited scope. Another problem of the method is that it is generally not easy to control the number of self-assembly, a factor that is the height of the cylindrical structures, because both the top and bottom faces can be involved in the self-assembly. A methodology is needed to inhibit the formation of higher face-to-face aggregation of aromatic molecules that produce tube-shaped complexes.

### 2.2. Double-Helix Chiral Cylindrical Molecular Complex

**Figure 5:**
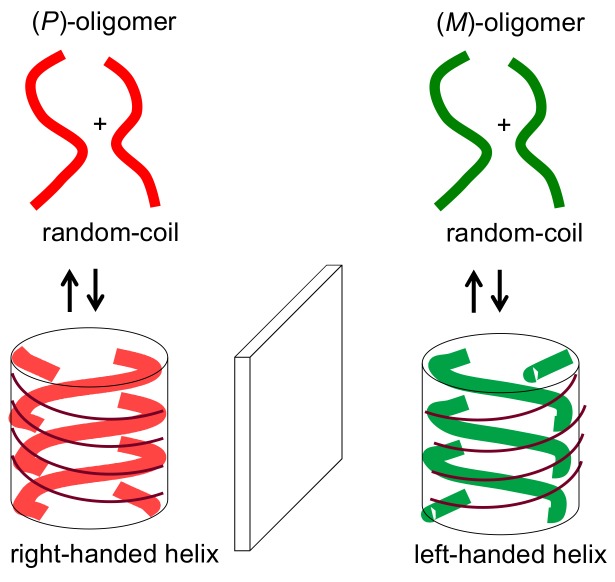
Formation of chiral double-helix cylindrical molecular complexes, shown in transparent blue.

An efficient method to synthesize cylindrical molecular complexes is double-helix formation by oligomeric molecules, which is the subject of this article (Figure 4). The method has advantages over a method using disk-shaped molecules. Structural diversity is high and various functional groups can be introduced at the terminal and side of the oligomers, which are located at the bottom faces and lateral face of a cylindrical structure, respectively. The height of the cylindrical structure can be controlled by the length of oligomers. When chiral oligomers are employed, double-helix chiral cylindrical structures are obtained and (*P*)- and (*M*)-double-helixes are formed from optically active (*P*)- and (*M*)-oligomers (Figure 5).

**Figure 6:**
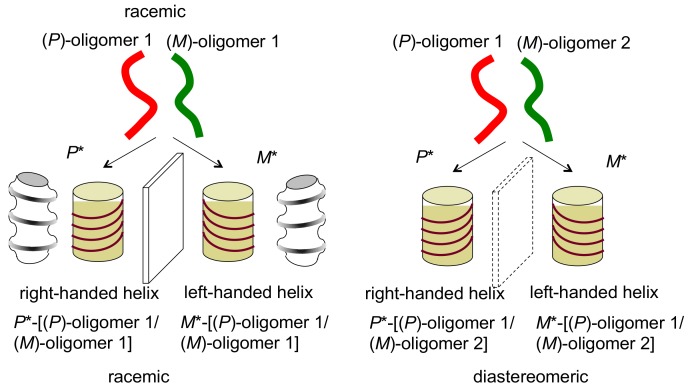
Hetero-double-helix chiral cylindrical molecular complexes with enantiomeric three-dimensional structures, which are derived from racemic oligomers providing enantiomeric structures and from slightly different structures of oligomers providing diastereomeric structures. Hetero-double-helixes are shown in transparent yellow.

When oligomers of the same structure aggregate, homo-double-helices are formed (Figure 5); when different structures aggregate, hetero-double-helices are formed (Figure 6). In this article, a homo-double-helix is shown in transparent blue and a hetero-double-helix in transparent yellow. Note that the hetero-double-helix can possess enantiomeric three-dimensional structures, termed P*- and M*-structures in this article, even when racemic oligomers are used, as shown in Figure 6a, because of the unsymmetrical twisting nature of hetero-double-helix structures. For example, racemic oligomers (*P*)-oligomer 1 and (*M*)-oligomer 1 can provide right-handed hetero-double-helix P*-[(*P*)-oligomer 1/(*M*)-oligomer 1] and left-handed hetero-double-helix M*-[(*P*)-oligomer 1/(*M*)-oligomer 1], which are enantiomeric. A mixture of slightly different structures of oligomers (*P*)-oligomer 1 and (*M*)-oligomer 2 can provide right-handed hetero-double-helix P*-[(*P*)-oligomer 1/(*M*)-oligomer 2] and left-handed hetero-double-helix M*-[(*P*)-oligomer 1/(*M*)-oligomer 2], which are diastereomeric (Figure 6). Such an example appears in the hetero-double-helix DNA, specifically B-DNA and Z-DNA. The synthetic systems are described in Section 4 and Section 5 of this article.

**Figure 7:**
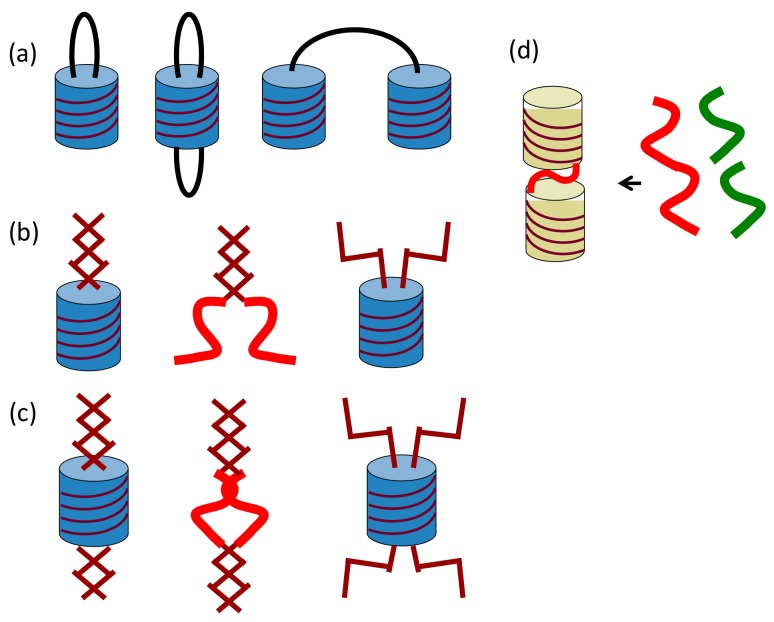
Linked cylindrical molecular complexes by covalent bond (**a**); single noncovalent bond domain (**b**); two noncovalent bond domains (**c**) and 1:2 double-helix complexes, where the length of oligomers are different (**d**).

Two or more double-helix chiral cylindrical molecular complexes can be linked by one and two covalent bonds, where the structures of the linking group can be modified (Figure 7a). Noncovalent bonds can also be employed for the linking groups, in which the chiral cylindrical molecular complex domain and the linker domain are either associated or dissociated (Figure 7b,c). These features are true for the 1:1 double-helix complexes derived from oligomers of comparable lengths, and, when the lengths are different, 1:2 double-helix complexes can be formed (Figure 7d).

### 2.3. Dynamics of Double-Helix Chiral Cylindrical Molecular Complex Formation

**Figure 8:**
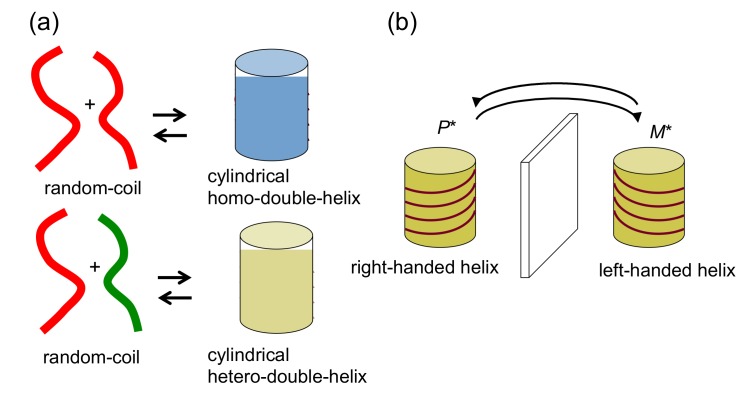
Interconversion between double-helix and random-coil (**a**) and interconversion between hetero-double-helix the enantiomeric three-dimensional structures (**b**).

Thus far, static structures of double-helix chiral cylindrical molecular complexes have been discussed but dynamics is another interesting subject with respect to two aspects (Figure 8): Structural changes between molecules or molecular complexes and concentration changes in response to changes in conditions are the thermodynamics aspect; and the mechanism of the structural changes are the kinetics aspect, which is also affected by changes in conditions.

Regarding the thermodynamic aspect (Figure 8), the formation of a double-helix chiral cylindrical molecular complex in solution can largely be affected by temperature, concentration and solvent type. In general, cooling and concentrating the solution promote association and vice versa. The solvent effect is also substantial and association is promoted in certain types of organic solvent. The effect is significant in aqueous solutions, where hydrophobic interactions are substantial. Thus, notable dynamics appears: sharp temperature responses due to changes in conditions, large enthalpic and entropic changes, ordinary and inverse thermo responses and interconversions between the chiral three-dimensional P*- and M*-molecular complexes.

The kinetic aspect during structural changes of double-helix chiral cylindrical molecular complexes is extremely complex and interesting. An example is self-catalysis, which is a phenomenon, in which a double-helix molecular complex catalyzes the reaction of random-coil molecules to become a double-helix. This is a molecular communication system, in which various unusual molecular events occur, such as thermal hysteresis and equilibrium crossing, which have been summarized in recent review articles [34,35,36,37,38].

Note that when the rate of the process to form double-helix chiral cylindrical molecular complexes is slower than the usual time-scale of minutes and hours, the kinetics of the processes can be followed by typical spectroscopic methods. In addition, the process can be perturbed by changing conditions during the process.

### 2.4. Self-Assembly of Double-Helix Chiral Cylindrical Molecular Complexes

**Figure 9:**
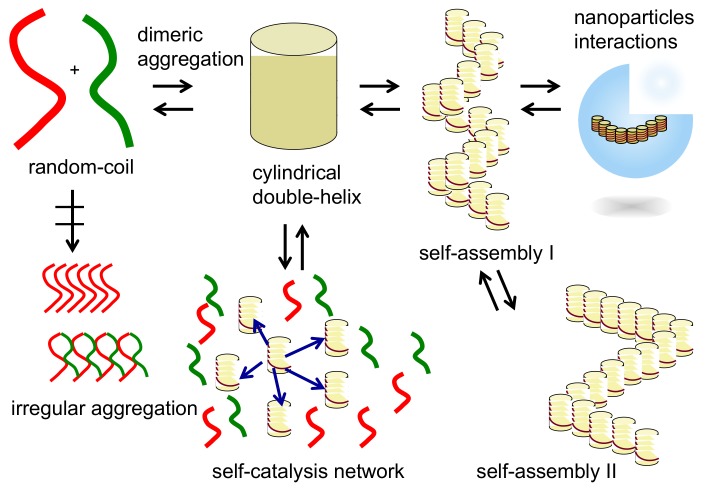
Structure changes of cylindrical molecular complexes of double-helix at the molecular level to self-assembly level in multiple steps manner.

Double-helix chiral cylindrical molecular complexes are capable of forming self-assembly materials by intercomplex interactions and provide heterogeneous liquid-solid materials, which are the subject of this article (Figure 9). These may be due to the broad and anisotropic surface and also to chirality. Various structures form from nanometer to centimeter sizes of materials. Self-assembly materials of subnano- to micrometer sizes were characterized by spectroscopy and microscopy, which are determined to be fibrils, fibers, bundles and vesicles. The structures of heterogeneous liquid-solid materials of micrometer to meter sizes were analyzed by microscopy, mechanical and visual methods, which were determined to be gels, liotropic liquid crystals and emulsions.

When molecular self-assembly occurs in one direction, fibrils are formed, which can aggregate to form fibers and bundles. When self-assembly occurs in two directions, sheets are formed, which can fold and assemble to form higher-order three-dimensional structures such as tubes and vesicles. When self-assembly occurs on flat and curved surfaces of solids, monomolecular layers are formed. Note that dissociated oligomers generally do not the self-assemble, which indicates an important role of the chiral cylindrical molecular complex structure in self-assembly. Chirality in cylindrical molecular complexes enables chiral recognition by the self-assembly.

### 2.5. Dynamics in Self-Assembly of Double-Helix Chiral Cylindrical Molecular Complexes

Dynamics in the self-assembly formation by the double-helix chiral cylindrical molecular complexes is another subject of this article (Figure 9). Regarding the thermodynamic aspect, self-assembly materials change their structures between dissociated states in response to changes in conditions. The relative thermodynamic stability of double-helix self-assemblies and dissociated states is largely affected by conditions. Structure changes between two self-assemblies I and II with ordered structures also appear (Figure 9), which can be useful compared with the changes between self-assembly materials and dissociated state, because two ordered structures are maintained during the switching, as exemplified in the mechanical movement of artificial muscles. In the Section 5 and Section 6, such examples are shown by the transition of self-assembly gels to vesicles; changes between self-assembly gels and liotropic liquid crystals; self-assembly gel shrinkage.

Regarding the kinetic aspect, the formation of heterogeneous liquid-solid materials by self-assembly in general involves multistep complex phenomena, in which many intermediates are formed. Examples are provided in this article such as liotropic liquid crystal formation, chiral symmetry breaking with self-assembly gel formation, discontinuous fibril film formation, mechanically induced self-assembly gel formation and silica nanoparticle precipitation. A strong Cotton effect and large changes in CD spectra appear accompanied by structure changes between double-helix and random-coil in helicene oligomers as well as during self-assembly. These spectral changes are monitored to determine complex kinetic processes in liquid-solid heterogeneous materials systems.

In this article, we discuss the double-helix formation by chiral helicene oligomers providing chiral cylindrical molecular complexes, which form diverse self-assembly materials in solution, heterogeneous liquid-solid materials. The conditions largely affect the structures and properties of self-assemblies. Dynamic properties in structural changes are discussed from the thermodynamic and kinetic aspects. In Section 3 and Section 4, the formation of chiral cylindrical molecular complexes by dimeric association of the helicene oligomers is shown to proceed by face-to-face interactions and double-helix formation. In Section 5 and Section 6, self-assembly of double-helix chiral cylindrical molecular complexes is discussed with regard to gelation and liotropic liquid crystals formation. In Section 7 and Section 8, self-assembly at solid surfaces is described. In Section 9, self-catalytic reactions involving the spatial homogeneous-heterogeneous transition in dispersed solution are described.

## 3. Double-Helix Chiral Cylindrical Molecular Complexes

Helical structures are ubiquitous in nature and appears in various materials from the nanometer scale at the molecular level to the centimeter scale at the bulk level. Examples are double-stranded DNA and α-helix proteins at the molecular level and chains of morning glories at the bulk level. Regarding synthetic molecules, however, helical structures are not common. Helicenes are an interesting group of synthetic aromatic molecules with a helical structure and have been known since the 1950s. Their properties have not been well examined, although many syntheses are reported [39,40,41,42,43,44,45,46,47,48,49,50,51]. On the basis of a large-scale synthesis of 1,12-dimethylbenzo[*c*]phenanthrene developed in this laboratory, we have studied the synthesis and function of helical substances using helicenes as building blocks [34,35,36,37,38] Helical substances treated here range from molecules to oligomers, molecular complexes and self-assembly materials, each of which exhibited notable properties and functions.

Several cyclic helicene oligomers have been developed and trimers form dimeric aggregates in solution, which are cylindrical molecular complexes by face-to-face aggregation. A number of acyclic helicene oligomers have also been developed and form double-helices in solution, which are another group of cylindrical molecular complexes. Double-helix molecular complexes self-assembled to form various heterogeneous liquid-solid materials. A notable feature of helical molecules is their chirality and right-handed and left-handed helical structures, which exhibit various chiral phenomena.

### 3.1. Face-to-Face Dimeric Aggregation of Cyclic Ethynylhelicene Oligomers

**Figure 10:**
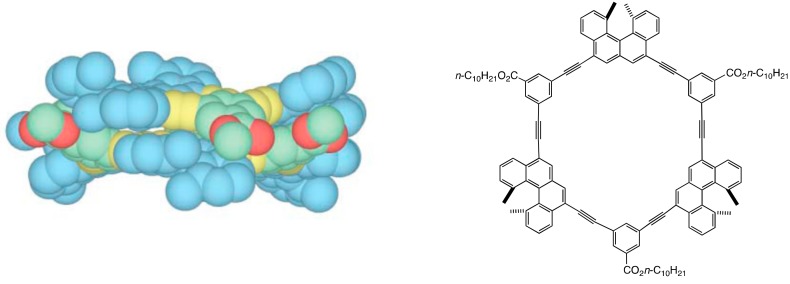
Calculated structure of dimeric (*P*)-[3 + 3]cycloalkyne. Reproduced from reference 52 by permission from ACS, Washington, DC, USA.

A series of cyclic ethynylhelicene oligomers have been synthesized, in which the helicenes and the *m*-phenylene spacers are linked by acetylenes, among which the trimers [3 + 3]cycloalkynes formed dimeric aggregates in solution (Figure 10) [52]. A (*P*)-[3 + 3]cycloalkyne with *D*_3_-symmetry possesses identical front and back surfaces. Cylindrical molecular aggregates are formed in solution and a structure arising from face-to-face aggregation was indicated by calculations. The upper and lower (*P*)-[3 + 3]cycloalkynes fit in the groves of the helicenes, which may make the other π-face not suitable to participate in further aggregation. Many examples of π-π stacking of aromatic compounds have been reported, which form high aggregates [32,33]. That [3 + 3]cycloalkynes form dimeric aggregates without forming high aggregates may be due to their chiral face structure.

**Figure 11:**
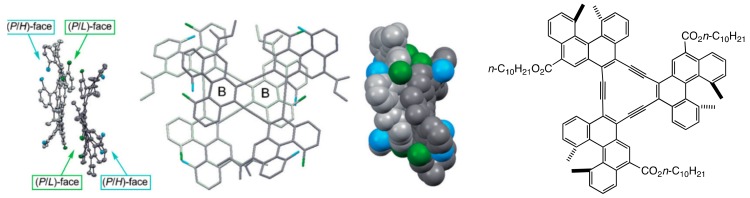
X-ray structure of dimeric (*P*)-[3]alkyne. Reproduced from reference 53 by permission from John Wiley & Sons, Hoboken, NJ, USA.

Chiral cylindrical molecular complexes were obtained from another helicene trimer [3]-alkyne, in which the helicene is connected by acetylenes without an *m*-phenylene spacer (Figure 11) [53]. The trimers again formed dimeric aggregates without forming high aggregates. In contrast to the (*P*)-[3 + 3]cycloalkyne, (*P*)-[3]alkynes with *C*_3_-symmetry have front and back surfaces, where three blade-like structures exhibit right-handed and left-handed chiral faces, providing front-right, front-left, back-right and back-left faces. Eight modes of face-to-face interactions appear in racemic (±)-[3]alkynes, among which the interactions between both front-right faces predominated in the solid state and in solution. Unlike the cylindrical molecular complexes formed by double-helices, which are discussed below, the cylindrical molecular complexes formed by disk-shaped molecules did not self-assemble in solution.

### 3.2. Double-Helix Formation by Ethynylhelicene Oligomers

**Figure 12:**
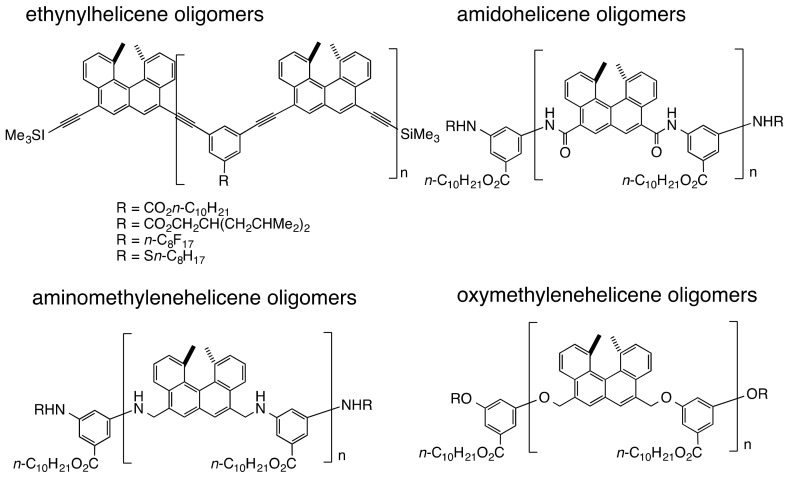
Chemical structures of acyclic helicene oligomers.

Acyclic helicene oligomers were synthesized, where helicenes and *m*-phenylenes were connected by acetylene, amide, reverse amide, sulfonamide, amino methylene and oxymethylene groups (Figure 12). To compare the structure and property of the oligomers with different numbers of helicenes, the oligomers were sequentially synthesized from monomers, dimers, trimers and higher oligomers [54,55]. All the compounds formed dimeric aggregates for oligomers above a certain number of helicenes.

**Figure 13:**
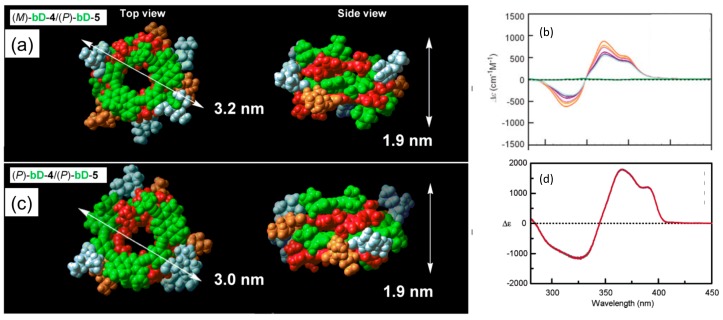
Calculated structures (**a**) and CD spectra (**b**) of hetero-double-helix derived from (*P*)-tetramer/(*M*)-pentamer; calculated structures (**c**) and CD spectra (**d**) of homo-double-helix derived from (*P*)-tetramer/(*P*)-pentamer.

The ethynylhelicene (*P*)-heptamer formed a homo-double-helix in chloroform, as determined by the following experiments [56,57,58,59]. An extremely strong Cotton effect appeared at ∆ε values exceeding 10^3^ cm^−1^ M^−1^, which indicated the formation of a highly ordered structure (Figure 13d); a decrease in UV-vis intensity caused by association indicated the involvement of π-π interactions; vapor pressure osmometry (VPO) indicated dimeric aggregation; ^1^H-NMR showed broadening and upfield shifts in the associated state, which indicated restricted molecular motion; heating dissociated the aggregates as shown by the weak Cotton effects, showing the random-coil state. The number of helicenes was critical for forming dimeric aggregates, where (*P*)-heptamers and longer oligomers aggregated in chloroform. Calculations on the (*P*)-tetramer/(*P*)-pentamer complex indicated a homo-double-helix structure with a chiral cylindrical structure, with a diameter of 3.0 nm and a height of 1.9 nm (Figure 13c). The (*P*)-tetramers formed a dimeric aggregate in trifluoromethylbenzene, which was consistent with the calculated homo-double-helix structure with three helicenes in one turn. A monomolecular layer of a homo-double-helix (*P*)-pentamer on a gold surface had a diameter of 3.0 nm as determined by QCM, consistent with the calculated diameter of 3.2 nm for the homo-double-helix: the 3.9 nm height of homo-double-helix (*P*)-pentamer attached to the phenylpropylthio group on a gold surface coincided reasonably with the calculated height of 1.9 nm for the (*P*)-pentamer/(*P*)-tetramer hetero-double-helix. The results indicate homo-double-helix formation by the ethnylhelicene oligomers with a chiral cylindrical structure.

Additional features supported homo-double-helix formation. Analysis of the thermodynamic parameters for the association of (*P*)-heptamers showed a large negative enthalpy change, ∆*H* = −98 kJ mol^−1^, which indicated very strong noncovalent bond interactions between the oligomers. A large negative entropy change ∆*S* = −0.33 kJ mol^−1^ K^−1^, was obtained, which indicated highly restricted molecular motions in the aggregates.

Structures of chiral cylindrical molecular complexes can be diversely modified by attaching various groups. In general, side chains were introduced at *m*-phenylene spacers, located at the lateral positions of the cylindrical structure to increase solubility [59] The lateral side chains affect the stability of homo-double-helices and perfluorooctyl and decyloxycarbonyl groups considerably increased the stability of homo-double-helices while the decylthio group destabilized them. For the terminal positions of the oligomers to be modified, they must be located at the axial positions of the cylindrical molecular complexes.

### 3.3. Thermo Response of Ethynylhelicene Oligomers

**Figure 14:**
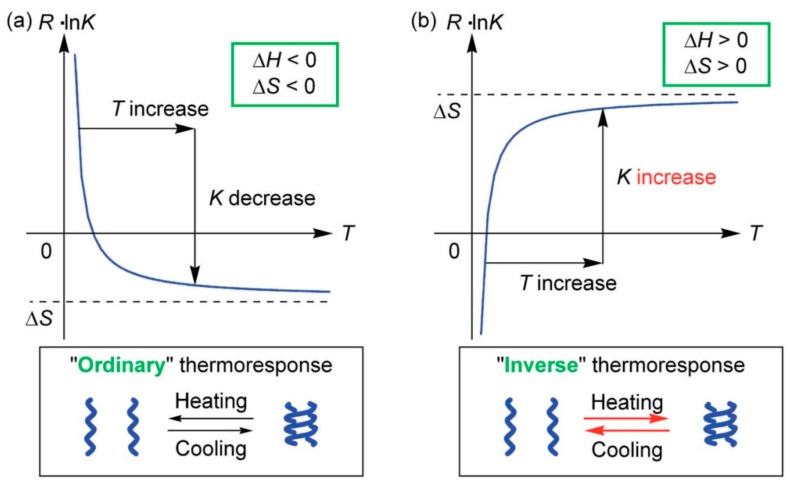
Ordinary thermo responses (**a**) and inverse thermo responses (**b**) shown by the plots of *RT*ln*K* against association constant *K*, in which the relation is shown by free energy, ∆*G* = −*RT*ln*K* = ∆*H* − *T*∆*S*.

Ethynylhelicene oligomers associate on cooling and dissociate on heating, which is an ordinary thermo response [56,57,58,59] All the molecules are homo-double-helices at low temperatures and are random-coils at high temperatures. These changes occurred for small temperature changes, for example, over 50 K in the case of the ethynyhelicene (*P*)-heptamer. This thermo response is explained by the large negative enthalpy change ∆*H* and large negative entropy change ∆*S* of the association reaction (Figure 14a). The large negative enthalpy change ∆*H* is derived from strong binding to form homo-double-helix and the large negative entropy change ∆*S* is derived from substantially reduced molecular motion by the formation of a compact structure of homo-double-helix. When the absolute value of ∆*H* is large, temperature dependences of the association constant *K* are substantial, because of *R*ln*K* = −∆*H*/*T* + ∆*S*, where *R* and *T* are the gas constant and absolute temperature, respectively.

The solvent effect is substantial and homo-double-helix formation is promoted in hard aromatic solvents such as trifluoromethylbenzene and *m*-difluorobenzene, whereas dissociation predominated in soft aromatic solvents such as iodobenzene and bromobenzene as determined by the hard-soft acid-base (HSAB) principle. The homo-double-helix formation of the ethynylhelicene oligomers in solution is sensitive to changes of conditions.

### 3.4. Inverse Thermo Response of Ethynylhelicene Oligomer with Terminal PEG Groups

Homo-double-helix chiral cylindrical molecular complexes were modified at the axial positions by introducing groups at the terminal positions of oligomers. Introduction of hydrophilic poly(ethylene oxide) (PEG) groups enhanced solubility in aqueous solutions, characterized by an inverse thermo response, that is cooling induced dissociation and heating association [60]. On the basis of the discussions of ordinary thermo responses, the inverse thermo response was considered to involve positive ∆*H* and ∆*S* values of the association reaction, which, however, is counter-intuitive, because the association reaction is endothermic and entropy increasing (Figure 14b).

**Figure 15:**
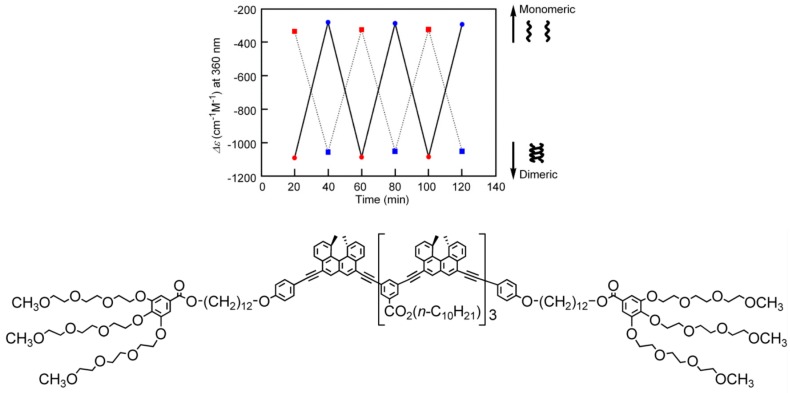
Ordinary thermo response (dotted lines) and inverse thermo response (solid lines) shown by ∆ε (360 nm)/time profiles at temperature switching between 5 and 60 °C shown in blue and red circles, respectively. Chemical structure of a PEG (*P*)-tetramer is also shown.

The ethynylhelicene (*M*)-tetramer with six terminal PEG groups showed an inverse thermo response in an aqueous solvent system of acetone/water/triethylamine (1:2:1) (Figure 15). DLS analysis confirmed that no higher aggregates were formed under these conditions and the inverse thermo response was a molecular event in dilute solution. The thermodynamic parameters for the association reaction were ∆*H* = +240 kJ mol^−1^ and ∆*S* = +0.92 kJ mol^−1^ K^−1^ both positive, which is unusual.

**Figure 16:**
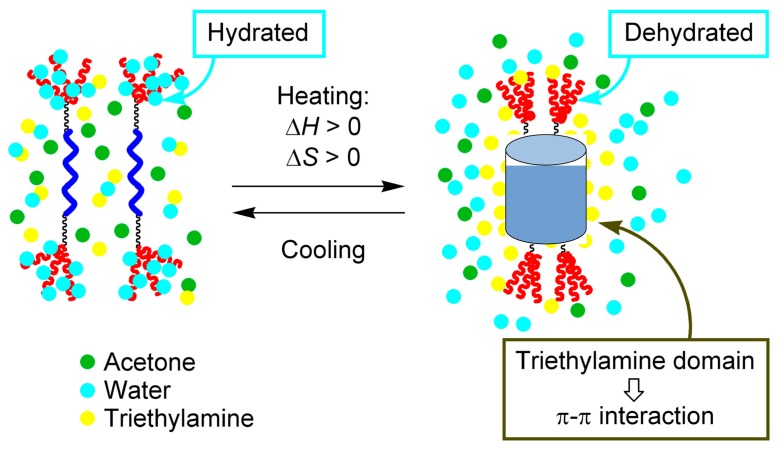
Possible mechanism of inverse thermo response.

A mechanism for the inverse thermo response based on hydration and dehydration at the PEG groups during cooling and heating, respectively, was considered (Figure 16). At a low temperature, the PEG groups, being hydrophilic, are hydrated by water molecules and the (*P*)-tetramer in random-coil state is dissolved in the aqueous solvent. As temperature increases, the PEG groups become hydrophobic and are dehydrated, which induces water molecules surrounding the hydrophobic group to form an ordered structure. To reduce the ordered water cluster domain, which is thermodynamically unfavorable, the (*P*)-tetramer associates in solution. Then, heating induces the association reaction, which is endothermic with a positive ∆*H*. The properties of water clusters outweigh the properties the homo-double-helices to dissociate because of the temperature increase.

Another feature that must be considered is the dimeric aggregate formation without polymeric aggregation. In general, hydrophobic organic molecules in aqueous solution form polymeric aggregates, as exemplified by lipid bilayers and micelles. This behavior is due to the minimization of the hydrophobic surface area in water, which reduces the extent of the ordered water cluster domain owing to hydrophobic interactions. The dimeric aggregate formation in this system may be explained by the lower critical solvent temperature (LCST) of the triethylamine/water system, which is homogeneous at low temperatures and phase-separated at high temperatures. When temperature increases, triethylamine clusters are formed, which incorporate the hydrophobic (*P*)-tetramers and promote homo-double-helix formation by the π-π interactions. This system must be considered with regard to the structure of molecules and solvent clusters.

### 3.5. Thermo Response of Linked and Multi-Domain Ethynylhelicene Oligomers

Homo-double-helix formation by linked ethynylhelicene oligomers in organic solvents was substantially affected by the nature of linking groups. When a flexible linker such as a hexadecamethylene group was employed to connect two ethynylhelicene (*P*)-hexamers, intramolecular homo-double-helices were formed [59] This behavior is ascribed to entropic factors, for which intermolecular homo-double-helix formation is unfavorable.

The linking can involve noncovalent chemical bonds. Bi-domain (*P*,*P*)-oligomer were developed, which contained an ethynylhelicene (*P*)-heptamer domain and an amidohelicene (*P*)-tetramer domain [61,62] The amidohelicene oligomers were capable of forming dimeric aggregates, although their aggregation property was different from that of ethynylhelicene oligomers: They aggregated in nonpolar solvents and disaggregated in polar solvents such as THF and DMSO; aggregation is insensitive to temperature changes. Heating the (*P*,*P*)-oligomer in nonpolar aromatic solvents induces dissociation at ethynylhelicene domains, while aminohelicene domains remained associated.

**Figure 17:**
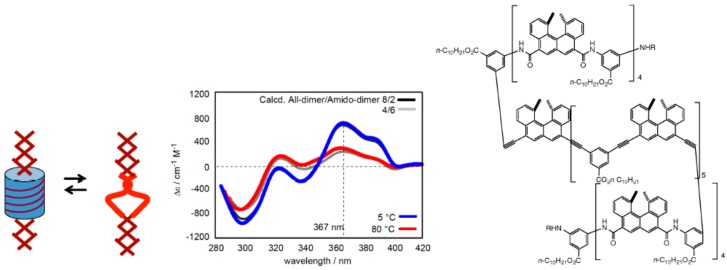
Expand and shrink of a tri-domain (*P*,*P*,*P*)-oligomer as shown by CD spectra. Chemical structure of tri-domain (*P*,*P*,*P*)-oligomer is also shown.

A tri-domain (*P*,*P*,*P*)-oligomer was developed, in which ethynylhelicene (*P*)-pentamer was connected by two amidohelicene (*P*)-tetramers at both terminals (Figure 17). In nonpolar solvents, a middle chiral cylindrical molecular complex domain associated and dissociated during cooling and heating, which likely caused expansion and contraction, because of the rigid aggregate structure in the amidohelicene domain [63].

### 3.6. Double-Helix Formation by Aminomethylenehelicene and Oxymethylenehelicene Oligomers

Aminomethylenehelicene oligomers were developed, in which the acetylene moieties in ethynylhelicene oligomers were substituted by aminomethylene groups (Figure 12) [64,65]. The aminomethylenehelicene oligomers formed homo-double-helix in solution, which associated and dissociated on cooling and heating. The homo-double-helix formation was shown by the very high CD intensities; reduced intensity by UV-vis, broadening of ^1^H-NMR absorptions, dimeric aggregation by VPO, similarity in the CD spectra shape with ethynylhelicene oligomers, helincene-number dependence and relatively large negative ∆*H* = −70 kJ mol^−1^ and ∆*S* = −0.18 kJ mol^−1^ K^−1^ values by association reaction of (*M*)-pentamer. Oxymethylenehelicene oligomers, where the nitrogen atoms in the aminomethylenehelicene oligomers were substituted with oxygen atoms, also formed homo-double-helix in solution (Figure 12) [66].

Homo-double-helixes were formed by helicene oligomers in solution without forming higher aggregates. The thermodynamic aspect of their dynamics has been discussed above and with regard to the kinetic aspect the structural changes are generally very rapid on a time scale of less than 1 min.

## 4. Hetero-Double-Helix Cylindrical Molecular Complexes

Compared with the homo-double-helix formation already described, hetero-double-helix formation is a complex phenomenon, in which the association must occur between two different suitable oligomers. In addition, the hetero-double-helices must be much stronger than those to form homo-double-helices; otherwise, mixtures of homo- and hetero-double-helices are formed, which complicates properties. The helicene oligomers containing enantiomeric helicenes formed hetero-double-helices (Figure 6). A notable feature of this phenomenon is that the helicene numbers in the enantiomeric oligomers need not the same and the enantiomeric oligomers with different numbers of the helicenes can be employed and resulting combinations are called pseudo-enantiomers. Mixtures of pseudo-enantiomeric oligomers are very close to racemic but with slight imbalances and various combinations of pseudo-enantiomeric oligomers are available. That the hetero-double-helix is not racemic allows structural analysis by CD spectroscopy.

### 4.1. Hetero-Double-Helix Formation by Pseudo-Enantiomeric Ethynylhelicene Oligomers

Mixtures of the pseudo-enantiomeric ethynylhelicene oligomers formed hetero-double-helices in solution, which were stronger than homo-double-helices. The phenomenon was initially observed using a mixture of the ethynylhelicene (*P*)-pentamer with perfluorooctyl side chains and the (*M*)-pentamer with decyloxycarbonyl, where the formation of 1:1 complex in chloroform was indicated by the Job plots (Figure 12) [64]. Hetero-double-helix formation was later confirmed using several combinations of oligomers, in which self-assembly of the hetero-double-helix, as is noted in the next section, was suppressed [65]. The combination of ethynylhelicene (*M*)-tetramer with branched decyloxycarbonyl side chain and (*P*)-pentamer with decyloxycarbonyl provided a dimeric aggregate as indicated by VPO. Calculated structure of the hetero-double-helix indicated a cylindrical structure analogous to the homo-double-helix (Figure 13a). Various structural features on hetero-double-helices including CD spectra were similar to the homo-double-helices (Figure 13b). The hetero-double-helix derived from a single combination of oligomers had two enantiomeric structures as indicated by the inverted CD spectra, which revealed the presence of chiral three-dimensional structures of the hetero-double-helices (Figure 6).

Such hetero-double-helix formation predominating over homo-double-helices was also observed for cyclic-linked bisethynylhelicene (*M*)-tetramers as will be described later [27]. The cyclic (*P*)-oligomer formed homo-double-helices in solution and addition of 2 equivalents of the (*M*)-pentamer provided 1:2 hetero-double-helices, as indicated by VPO analysis.

The ααββ tetrameric aggregate was obtained by hetero-double-helix formation from a bi-domain (*P*,*P*)-oligomer and an (*M*,*P*)-oligomer as will be described later [62] Mixing two compounds in the dimeric aggregate state provided the ααββ tetrameric aggregate, as determined by VPO, where the hetero-double-helix formed at the ethynylhelicene domain.

### 4.2. Hetero-Double-Helix Formation from Pseudo-Enantiomeric Aminohelicene Oligomers

**Figure 18:**
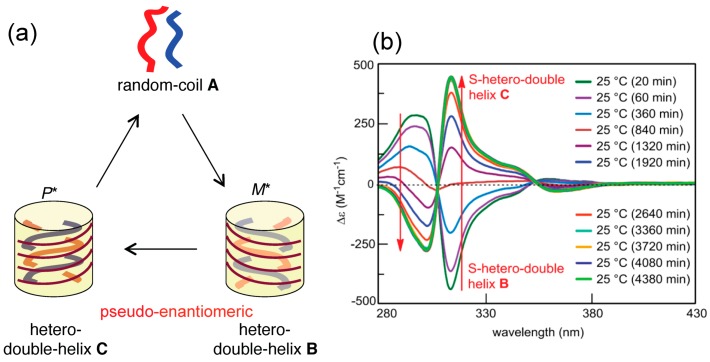
Inversion of chiral three-dimensional structures in the hetero-double-helix formation derived from aminomethylenehelicene (*P*)-tetramer and (*M*)-pentamer, which are indicated by the P*- and M*-structures (**a**); CD spectra for the **B**-to-**C** reaction are also shown (**b**).

Pseudo-Enantiomeric aminomethylenehelicene oligomers formed hetero-double-helices, which predominated homo-double-helices (Figure 12 and Figure 18) [66,67]. A mixture of a (*P*)-tetramer and an (*M*)-pentamer at 70 °C, as random-coils **A**, was cooled to 25 °C at a constant rate, which provided a metastable **A** solution. Hetero-double-helix **B** was formed with a strong negative Cotton effect at 315 nm within 1 h. When the solution was settled at 25 °C for 60 h, the CD spectrum inverted and provided another hetero-double-helix **C** with a strong positive Cotton effect at 315 nm. The inverted nature of the CD spectra of **B** and **C** revealed the apparent enantiomeric three-dimensional structures, although, to be precise, they are diastereomeric because of the use of the (*P*)-tetramer and (*M*)-pentamer (Figure 6). The results indicated that **B** with a chiral three-dimensional structure, tentatively termed M*, was inverted to **C** with the enantiomeric structure P*. The hetero-double-helix structures of **B** and **C** were determined from the high CD intensity, reduction in intensity by UV-vis, broadening of ^1^H-NMR absorptions, dimeric aggregation by VPO, similarity in shape of CD spectra with the ethynylhelicene oligomers, helicene-number dependence, 1:1 complexation as shown by the Job plots experiment and the enantiomeric form of the hetero-double-helix derived from a single combination of oligomers.

The **B**-to-**C** reaction is an example of the chirality inversion of hetero-double-helices, where **C** is thermodynamically more stable than **B** (Figure 6). The inversion of enantiomeric structures with a relatively large energy barrier was slow and occurred on the time-scale of hours. The mechanism could be either intramolecular inversion of three-dimensional structures or intermolecular dissociation and association. Heating **C** regenerated the dissociated **A**, which constructed three-states one-directional structure change with a single heating.

Slow hetero-double-helix formation often occurs on the time-scale of hours, which compares with rapid homo-double-helix formation. Then, the kinetics of the processes can be monitored by spectroscopic methods and perturbations introduced during the process caused interesting nonequilibrium thermodynamic phenomena, which were summarized in a recent article [35,36].

## 5. Self-Assembly Gel Formation by Hetero-Double-Helix Cylindrical Molecular Complexes

Gels are semi rigid heterogeneous liquid-solid materials, which do not flow. Self-assembly gels, where small particles or molecules self-assemble to form fibrous structures incorporating large amounts of solvent molecules [68,69,70], have attracted attention, because gel structure and property can be tuned by altering molecular structures. Two-component self-assembly gels have been developed, in which gelation occurs only in the presence of molecules with two different structures. The scope of self-assembly gel formation, however, is not broad and modifications of molecular structures often causes the loss of gel formation ability, probably because interactions between the molecules are weak. In contrast, chiral cylindrical molecular complexes exhibit a broad scope of self-assembly gel formation, which may be due to strong intercomplex interactions. The fact that molecular structures can be modified is an important advantage.

### 5.1. Self-Assembly Two-Component Gels of Heterogeneous Liquid-Solid Materials Derived from Pseudo-Enantiomeric Ethynylhelicene Oligomers

**Figure 19:**
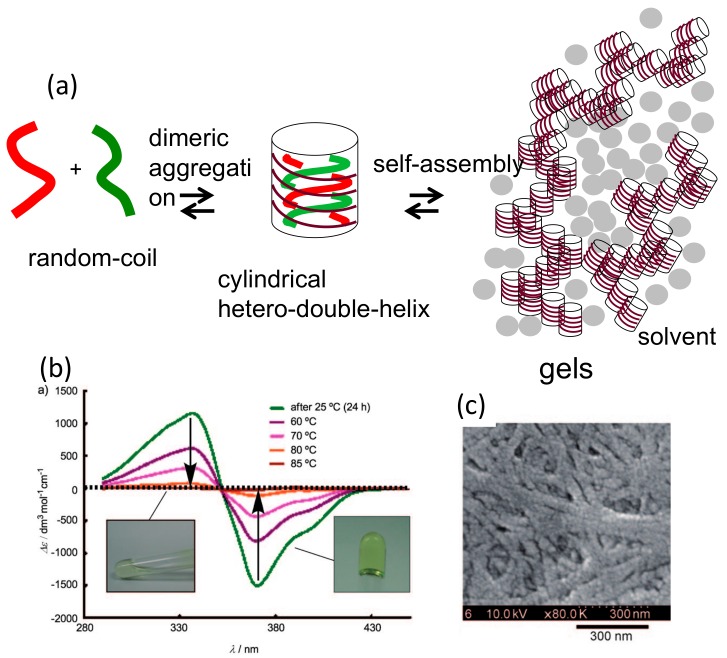
Self-assembly gel formed by a hetero-double-helix (**a**) shown by CD spectra (**b**) and TEM image (**c**). Reproduced from reference 71 by permission from John Wiley & Sons.

Hetero-double-helices derived from ethynylhelicene oligomers formed self-assembly gels (Figure 19a) [71]. A pseudo-enantiomeric mixture of the ethynylhelicene (*P*)-tetramer and (*M*)-pentamer in toluene at 70 °C, which are random-coils, was cooled to 25 °C and a metastable random-coils solution was formed. Then, slow hetero-double-helix formation and self-assembly occurred, as indicated by the appearance of a strong negative Cotton effect at 380 nm and the increase in the viscosity of the solution (Figure 19b). TEM analysis indicated formation of a fibrous structure (Figure 19c).

Various combinations of pseudo-enantiomeric oligomers form gels, in which both were trimers and longer oligomers. This behavior showed the broad scope of this method (Table 1). Self-assembly occurs only under conditions, when hetero-double-helices forms and were not in random-coil state. The anisotropic nature of hetero-double-helix chiral cylindrical structures facilitated the elongation and thickening of the self-assembly to form fibrils, fibers and bundles, which then formed gels.

**Figure 20:**
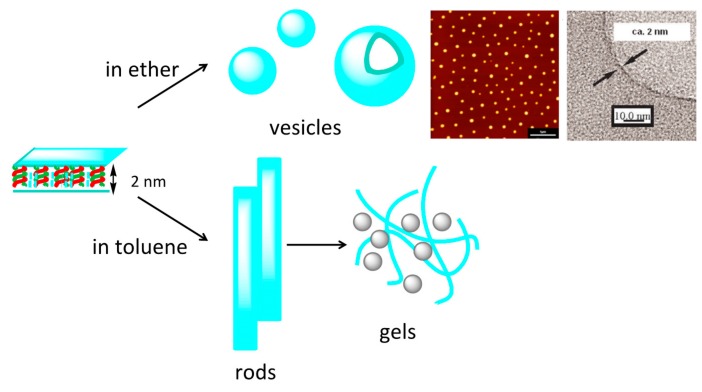
Effects of solvents on the morphology of self-assembly materials derived from hetero-double-helices (*P*)-pentamer/(*M*)-tetramer.

When the solvent was changed, different self-assembly materials were formed. Vesicles were formed in diethyl ether and fibrous structures were formed in toluene [71]. The thickness of the membrane 2 nm in vesicles were close to the height of the hetero-double-helix (Figure 13), suggesting that the lateral interactions of the chiral cylindrical molecular complex formed single molecular membrane (Figure 20). The different morphologies can be ascribed to the different folding modes of the single molecular membrane; folding into tubes in toluene and balls in diethyl ether. The vesicles could be converted to gels by changing the solvent and by heating, which is an example of a structure changes between ordered self-assembly materials.

**Figure 21:**
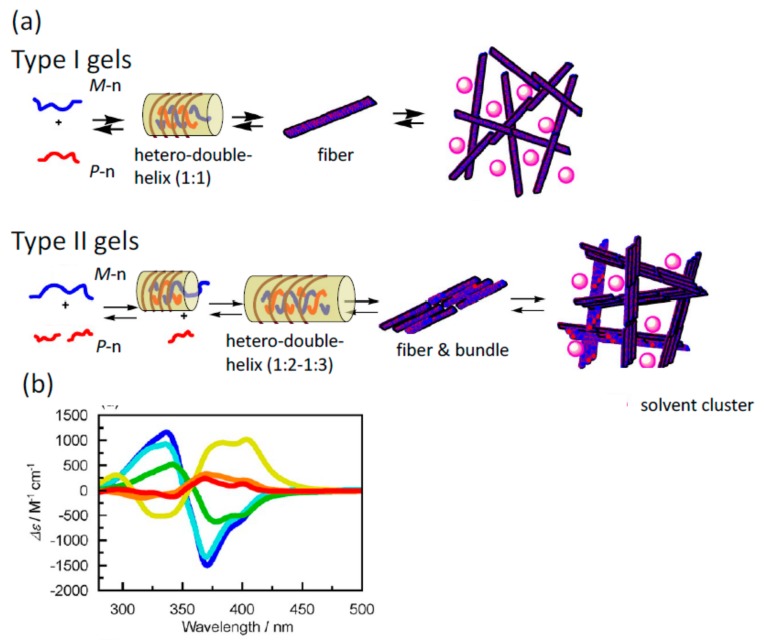
Type I and Type II gels in the pseudo-enantiomeric ethynylhelicene oligomers (**a**) shown by CD spectra (**b**). Reproduced from reference 72 by permission from ACS.

An advantage of this method to form two-component self-assembly gel is that the gel structures and properties can be tuned by the structures of helicene oligomers, which involve the number of helicenes, the side chain structure, the linker structure and the employment of multi-domain oligomers. In addition, different combinations of two pseudo-enantiomeric oligomers provide different structures and properties of self-assembly gels (Table 1 and Figure 21) [72] In accordance, depending on the numbers of two pseudo-enantiomeric oligomers, two types of gels were formed: combinations of pseudo-enantiomeric oligomers with comparable numbers of helicenes (Type I) and combinations of pseudo-enantiomeric oligomers with larger differences in the number (Type II). Type I gels showed relatively lower minimal gelation concentrations (MIC) than Type II gels; the Cotton effect at 380 nm was characteristically negative for Type I gels and positive for Type II gels, despite using (*M*)-oligomers for the longer component; the stoichiometries were 1:1 for Type I gels and 1:2 to 1:3 for Type II gels; Type I gels were more viscous than Type II gels. The formation of enantiomeric structures of gels as indicated by CD spectra may be due to the enantiomeric three-dimensional structures of hetero-double-helixes (Figure 21b).

**Figure 22:**
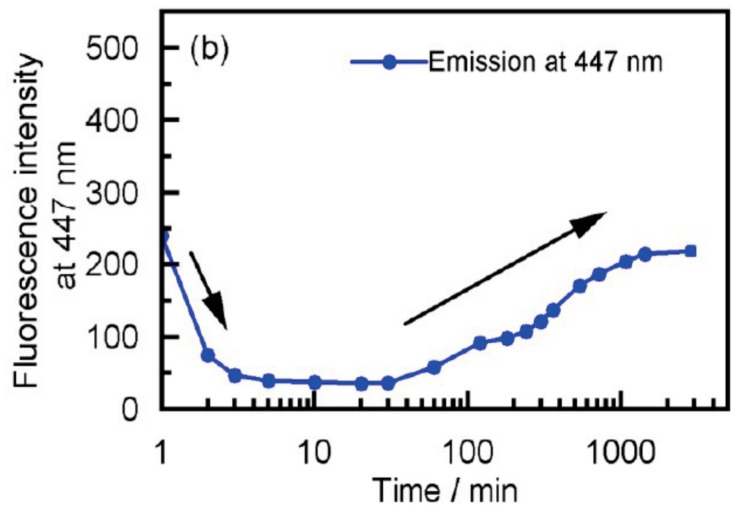
Formation of type I gels shown by fluorescence/time profiles. Reproduced from reference 72 by permission from ACS.

The dynamics in the self-assembly gelation in terms of kinetic aspects appears to be complex, as indicated by the fluorescence/time profiles (Figure 22). The fluorescence/time profile shows decrease followed by an increase.

### 5.2. Gels Formed by Bi-Domain Oligomers

Bi-domain helicene oligomers also formed self-assembly gels derived from the hetero-double-helices, in which different properties of two domains are combined to exhibit a novel property. This methodology is referred to as synthesis of function.

**Figure 23:**
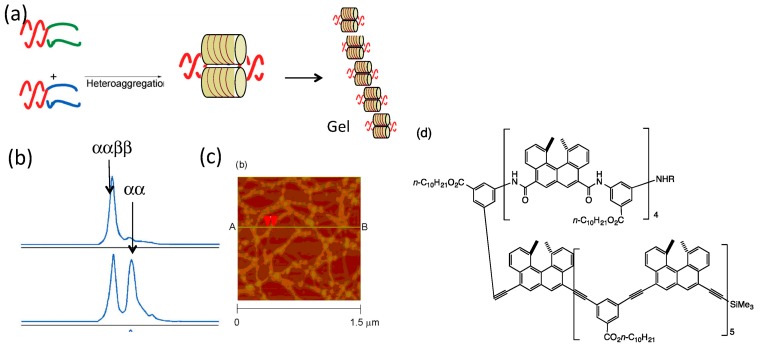
Formation of ααββ tetrameric aggregates derived from bi-domain (*P*,*P*)- and (*P*,*M*)-compounds (**a**); HPLC profiles (**b**) and AFM images (**c**) of gels are shown. Chemical structure of bi-domain (*P*,*P*)-oligomer is also shown (**d**).

An ααββ tetrameric aggregate was obtained by hetero-double-helix formation by the bi-domain (*P*,*P*)-oligomer and (*M*,*P*)-oligomer (Figure 23), which were the α and β subunits, respectively and which contained the ethynylhelicene (*P*)/(*M*)-pentamer domain and amidohelicene (*P*)-tetramer domain [62]. The (*P*,*P*)-oligomer and (*M*,*P*)-oligomer formed a dimeric aggregate at the amidohelicene domain and mixing provided the ααββ tetrameric aggregate by hetero-double-helix formation at the ethynylhelicene domain. Biological proteins form ααββ tetrameric aggregates and it is interesting that such higher-order structures can be constructed without forming simple αβ dimeric aggregates. A method of synthesis was developed using the bi-domain oligomers, on the basis of the stronger association of hetero-double-helix than of homo-double-helix.

At higher concentrations, the ααββ tetrameric aggregate formed gels by the self-assembly at the hetero-double-helix moiety, which resulted in a four-component self-assembly gel (Figure 23c). By heating and cooling, a reversible sol-gel transition occurred as a result of the dissociation and association of the hetero-double-helices.

**Figure 24:**
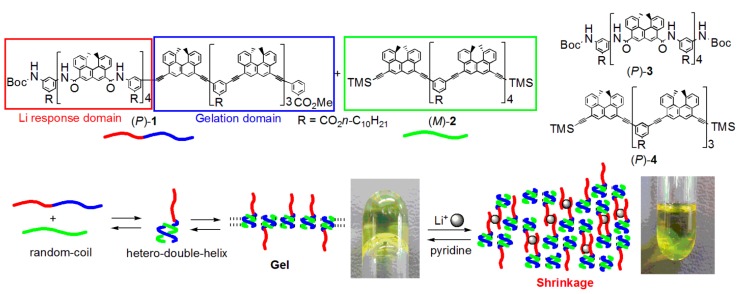
Synthesis of function by bi-domain (*P*,*P*)-compound and (*M*)-tetramer upon addition and removal of lithium salt.

Another example of the synthesis of function by multi-domain oligomers was shown in the reversible shrinkage of self-assembly gels induced by addition and removal of lithium cation (Figure 24) [73]. A mixture of the bi-domain (*P*,*P*)-oligomer and the ethynylhelicene (*M*)-pentamer formed self-assembly gel in pyridine, which reversibly dissociated upon heating and associated upon cooling. When lithium perchlorate was added, the gel shrunk to 1/3 of its original volume, owing to the interactions of lithium cations with amidohelicene domains. When the sol phase was removed and pyridine was added, the gel expanded, regaining its original size. The response to lithium cations at the amidohelicene domains and gelation at the ethynylhelicene domains together caused reversible gel shrinkage, thereby illustrating the synthesis of function by multi-domain oligomers. This behavior is another example of structural changes between two ordered self-assembly materials.

An advantage of two-component gel formation using the pseudo-enantiomeric ethynylhelicene oligomers is diversity in the self-assembly materials, in which various combinations of the oligomers can be employed. The strong tendency of the hetero-double-helix cylindrical molecular complexes to form self-assembly gels enables various structural and functional modifications without losing gelation property.

### 5.3. Chiral Symmetry Breaking Accompanied by Self-Assembly Gel Formation

Chiral symmetry breaking is a phenomenon, in which symmetric chiral state is transformed into unsymmetrical chiral state; this involves fluctuation to form one of the enantiomeric structures [74,75,76,77,78] Along with a significance in basic science, its application to the production of optically active compounds has attracted attention because of its simple operation. Several examples of this phenomenon are known with self-assembly involving achiral organic compounds and racemic compounds under racemization conditions. The mechanisms, however, are not yet well understood. Chiral symmetry breaking by racemic organic molecules under nonracemization conditions is interesting, because diverse combinations of components are available as well as diverse conditions: Experiments can be conducted systematically by changing the ratio, mixing mode, or procedures in the mixing of the enantiomers.

**Figure 25:**
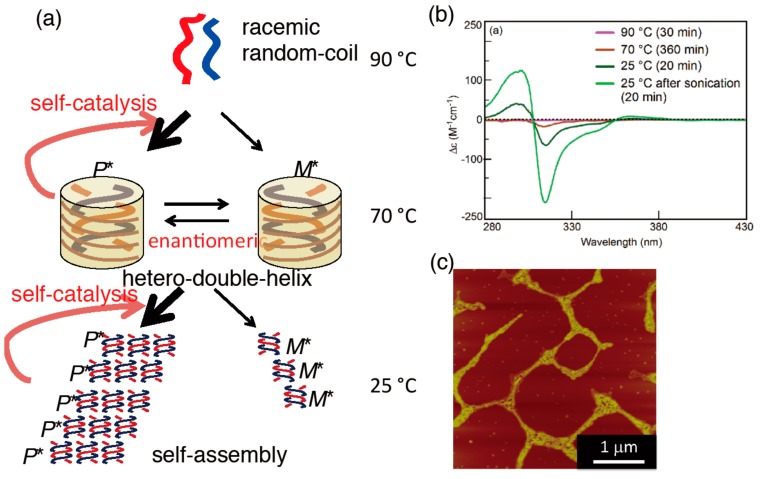
Schematic presentation of chiral symmetry breaking of racemic aminomethylehehelicene (±)-pentamer (**a**) as shown by development of CD spectra (**b**) and AFM images of resultant fibers (**c**). Reproduced from reference 79 by permission from John Wiley & Sons.

As noted previously, 1:1 mixture of the pseudo-enantiomeric aminomethylenehelicene (*M*)-pentamer and (*P*)-tetramer formed hetero-double-helices **B** and **C** with the enantiomeric three-dimensional structures (Figure 18). The racemic (±)-pentamer was then examined, which exhibited deterministic and stochastic chiral symmetry breaking [79]. The deterministic implies the formation of a single enantiomeric hetero-double-helix and self-assembled structures as shown by repeated experiments; stochastic implies the formation of enantiomeric structures at comparable ratio (Figure 25a). The system involved the formation of enantiomeric hetero-double-helices (Figure 6) and its self-assembly to form fibrils, during which chiral symmetry breaking occurred under nonracemization conditions.

Equal amounts of the (*P*)-pentamer and (*M*)-pentamer were weighed and were dissolved in toluene. The solution was heated to 90 °C to establish the random-coil state and then cooled to 70 °C, at which point the Cotton effect with small negative and positive values appeared at 315 and 303 nm, respectively (Figure 25b). Chiral symmetry breaking appeared during the formation of the enantiomeric structures of hetero-double-helix. On cooling to 25 °C, a self-assembly gel was formed and the Cotton effect was enhanced, indicating the chiral symmetry breaking under self-catalytic conditions (Figure 25c). Depending on the conditions, deterministic and stochastic chiral symmetry breaking appeared.

**Figure 26:**
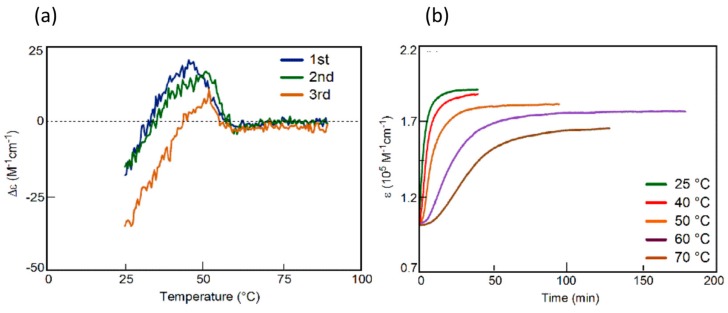
Kinetics in chiral symmetry breaking during hetero-double-helix and self-assembly gel formation shown by ∆ε/time profiles from repeated experiments (**a**) and ε/time profiles at different temperatures (**b**). Reproduced from reference 79 by permission from John Wiley & Sons.

Complex kinetics appeared in the chiral symmetry breaking, as shown by the ∆ε/time profiles from the constant cooling rate experiments and a sigmoidal curve in isothermal experiments (Figure 26).

Hetero-double-helix chiral cylindrical molecular complexes formed various self-assemblies in solution, owing to strong intercomplex interactions. Heterogeneous liquid-solid materials involving self-assembly solid phases then exhibited unique structures and dynamic properties, which can be tuned by altering the molecular structures and conditions. The complex kinetic of the homogeneous-heterogeneous transition are also noted.

## 6. Self-Assembly Lipotropic Liquid Crystal Formation by Chiral Cylindrical Molecular Complexes

Liotropic liquid crystals (LLC) are heterogeneous liquid-solid materials that are also anisotropic [80,81,82]. Both self-assembly gels and LCCs are formed from fibrous structures containing large amounts of solvent molecules. They can be differentiated by polarized optical microscopy (POM), which provides homogeneous images for the gels and birefringence for the LCC. These differences are ascribed to a random orientation of the fibers in the gels and an anisotropic arrangement at the macroscopic level on the order of micrometers to millimeters in the LLCs.

**Figure 27:**
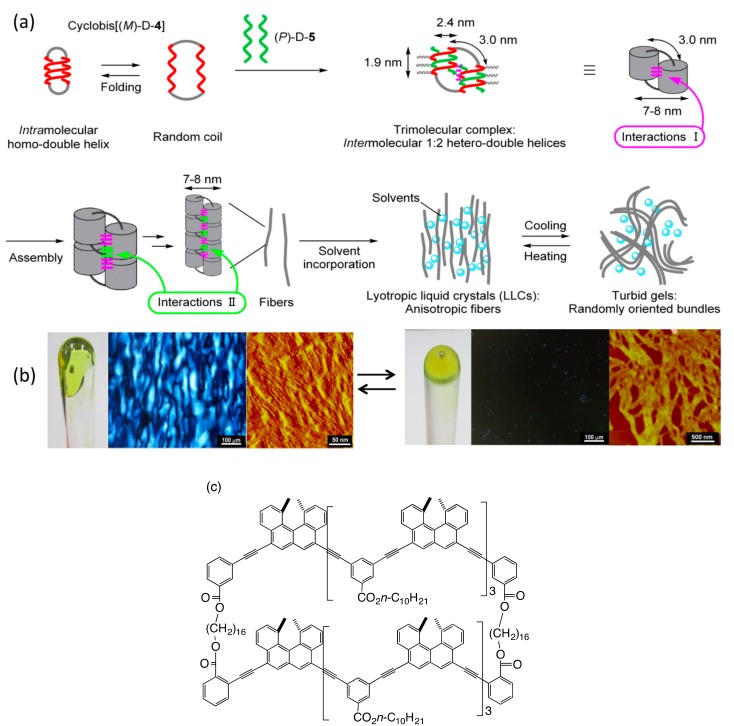
Schematic representation of a hetero-double-helix molecular complex and self-assembly of the cyclic ethynylhelicene bis[(*M*)-tetramer]/(*P*)-pentamer system (**a**); Visual, POM and AFM images of LLCs at 25 and −60 °C (**b**); The chemical structure of the cyclic bis[(*M*)-tetramer] is also shown (**c**).

Linked cyclic bis[(*M*)-tetramers] derived from an ethynylhelicene oligomer showed a structural change at the molecular level between an intramolecular double-helix and random-coils induced by cooling and heating (Figure 27) [83]. In the presence of an enantiomeric (*P*)-pentamer, trimolecular 2:1 complexes with a total molecular weight of over 10,000 daltons containing intermolecular hetero-double-helices was formed, which predominated over the intramolecular homo-double-helix.

At higher concentrations, the trimolecular complex self-assembled and formed LLC composed of anisotropically aligned fibers. A mixture of a linked cyclic bis[(*M*)-tetramer] and a (*P*)-pentamer in toluene gradually changed into a viscous fluid and POM showed birefringence with a poly-domain texture typical of the nematic LLC phase (Figure 27b). SAXS analysis indicated regular periodicity with a distance of approximately 9 nm perpendicular to the nematic director, which was ascribed to the width of mesogens with the surrounding solvent. AFM analysis of the LLCs showed that they partially aligned to form fibers of 7−8 nm diameter.

When the LLCs were cooled to −60 °C, their transparency decreased and viscosity increased, at which the LLCs changed into a turbid gel (Figure 27a). POM analysis showed the disappearance of birefringence and AFM analysis showed random oriented thick bundles 100–200 nm in width. The changes were reversible and the birefringence recovered when the mixture was warmed to 25 °C. This is a reversible change between two ordered self-assembled materials, which contrasts to generally known changes between LLCs and nonstructured isotropic liquids.

## 7. Solid Surface Self-Assembly by Chiral Cylindrical Molecular Complexes

Chemical reactions, conformational changes and aggregations of organic molecules at the liquid-solid interfaces are important phenomena in nature and exhibit different properties from that occur in solution [84,85,86,87] The differences can be ascribed to the interactions of molecules with the surface, higher concentrations of organic molecules on the surface and confined conformations of molecules on the surface. Less, however, is known about the chemical reactivities of organic molecules on a solid surface than those in solution. Homo- and hetero-double-helix chiral cylindrical molecular complexes self-assemble on solid surfaces and exhibit notable dynamics.

### 7.1. Homo-Double-Helix Formation on Gold Surface

**Figure 28:**
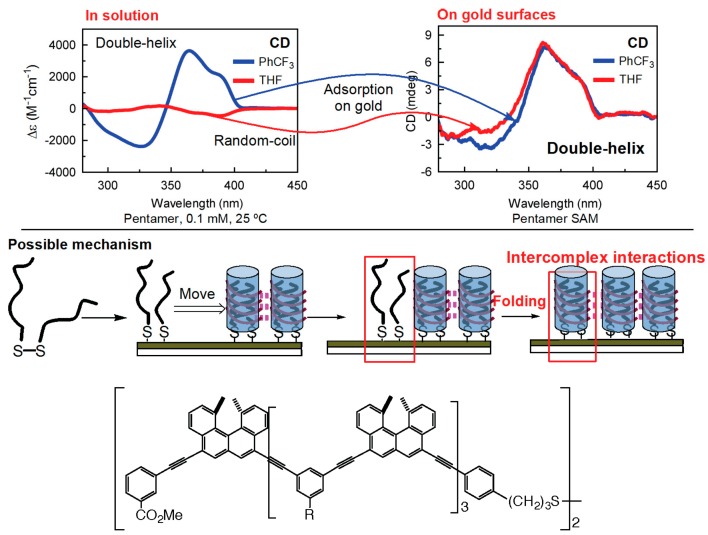
Formation of self-assembled monolayer (SAM) of double-helix (*P*)-pentamer disulfide on a gold surfaces. Chemical structure of the (*P*)-pentamer disulfide is also shown.

Ethynylhelicene (*P*)-pentamer disulfide formed a self-assembly monolayer (SAM) with homo-double-helix structure on a gold surface [88]. When a gold plate was immersed in a solution containing homo-double-helix (*P*)-hexamer disulfide, the homo-double-helix were grafted on the gold surface with a thickness of 3.9 nm as determined by quartz crystal microbalance (QCM) (Figure 28). From the average coverage, a double-helix occupied an area of 6.5 nm^2^ area with a diameter of 2.8 nm, which was consistent with the calculated size of the bottom face (Figure 13). The bottom face of the homo-double-helices were considered attached to the gold surface with dense packing. The structure of this SAM system can be monitored by CD, taking advantage of the extremely strong Cotton effect resulting from the homo-double-helix formation.

An interesting dynamic on the gold surface occurs when a gold plate is immersed in a solution containing random-coils of (*P*)-pentamer disulfide and SAM with a homo-double-helix structure is produced, indicating that the interactions of the sulfur groups with the gold surface induce the association. Dissociation did not occur by heating the homo-double-helices on the surface to 80 °C. The formation of homo-double-helices on the gold surface is ascribed to the strong intercomplex interactions between the chiral cylindrical molecular complexes. 

### 7.2. Formation of Fibril Films on Vesicle Surfaces

Fibrils and fibers form by self-assembly of organic molecules in solution and provides gels incorporating large amounts of solvent molecules. In contrast, fibril formation at a liquid-solid interface produces fibril films, which is related to biological events on the membranes, cells and also solid surfaces [89,90]. For example, formation of amyloid fibrils by the aggregation of peptides or proteins is considered to be related to Alzheimer’s, Parkinson’s and prion diseases. The surface adsorption of peptide and protein films is also a critical subject in relation to the biocompatibility of implantation materials and biofilm formation by microorganisms.

**Figure 29:**
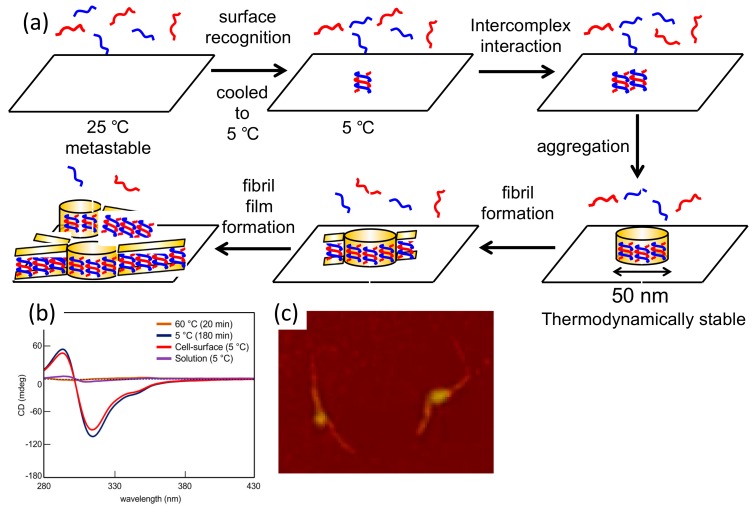
(**a**) Schematic presentation of fibril film formation shown by (**b**) CD spectra and (**c**) AFM image.

A pseudo-enantiomeric mixture of oxymethylenehelicene oligomers (Figure 12) formed hetero-double-helices at the liquid-solid interface of vesicles, which provided fibril films (Figure 29a) [91]. Discontinuous nucleation was characteristic of the dynamics of the fibril film formation. A 1:1 mixture of an (*M*)-hexamer and a (*P*)-pentamer (Figure 12) in trifluoromethylbenzene was heated to 60 °C in a quartz cell and cooled to 5 °C, which produced a metastable homogeneous solution with dissociated random-coils. When the solution was held at 5 °C, fibrils formed on the quartz cell surface, which self-assembled into fibril films (Figure 29b). In contrast, the oligomers remained dissociated in the solution phase.

It may be reasonable to consider the initial hetero-double-helix formation on the solid surface on the basis of the following observations: very strong negative Cotton effect at 316 nm; reduced UV-vis intensity; 1:1 complexation determined from Job plots; hetero-double-helix formation by related pseudo-enantiomeric aminomethylenehelicene oligomers (Figure 18); similar CD spectra with the hetero-double-helix of pseudo-enantiomeric aminomethylenehelicene oligomers; presence of the enantiomeric three-dimensional structures.

The fibril film was formed on different substrate plates and the amounts differed by a factor of 20 between poly(ethylene terephthalate) and aluminum. The result is consistent with the involvement of the solid surface in the formation of hetero-double-helices as well as their self-assembly.

With regard to the kinetic aspect of the fibril films formation, AFM analysis at early stages showed a number of flat and round particles with a uniform diameter of 50 nm (Figure 29c). Notably, short fibrils were formed from some particles, which indicated that the particles were the critical for initiating fibril formation. The ∆ε/time profiles of fibril film formation revealed a sigmoidal kinetics with a lag time.

A probable mechanism of fibril film formation follows (Figure 29a). At 60 °C, the (*P*)-pentamer and (*M*)-hexamer are in the dissociated state and produce a metastable random-coil solution upon cooling to 5 °C. Then, hetero-double-helices are formed on the solid surface, which self-assemble to particles 50 nm in diameter. The particles cease to grow, likely because of the formation of thermodynamically stable ordered structures, from which fibrils 50 nm width form. The intense CD signals and large changes accompanying homo-double-helix formation enable the structural analysis on the solid surface. The discontinuous nucleation is programmed in the molecular structures of the oxymethylenehelicene oligomers and is another example of dynamics between two ordered self-assembled structures.

### 7.3. Self-Assembly Gels Formed by Mechanical Stimulation

**Figure 30:**
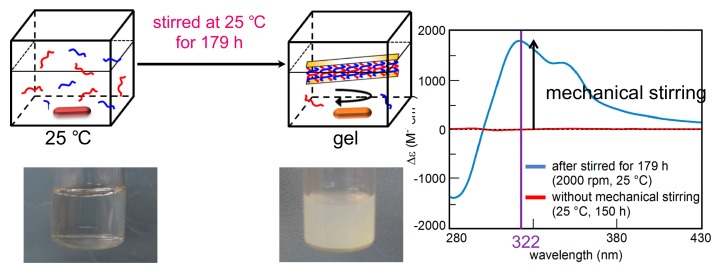
Formation of hetero-double-helices and self-assembly of oxymethylenehelicene (*M*)-hexamer and (*P*)-pentamer mixture in solution at 25 °C by mechanical stirring. The changes are shown by visual images and CD spectra.

Molecular responses to mechanical stimulations are an interesting subject, which has possible applications in mechanical switching, mechanical sensing and friction control [92,93,94,95,96,97,98,99,100]. Hetero-double-helix and self-assembly gels formation in pseudo-enantiomeric mixtures of the oxymethylenehelicene (*P*)-pentamer and (*M*)-hexamer occurred at 25 °C in response to mechanical stirring (Figure 30). This behavior is in contrast to the formation of fibril films on the solid surface at 5 °C (Figure 29) [101].

A trifluoromethylbenzene solution of a 1:1 mixture of the (*P*)-pentamer and (*M*)-hexamer was heated to 80 °C and cooled to 25 °C to produce a metastable homogeneous solution. The solution was mechanically stirred with an oval-shaped Teflon magnetic stirring bar at a rate of 2000 rpm (Figure 30). The solution became turbid and CD spectra with a positive Cotton effect at 322 nm was recorded, which indicated the formation of the hetero-double-helices and self-assembly. It was determined that fibril film was not formed on the vessel surface under these conditions. The inverted shapes of the CD spectra of the self-assembly gels and the fibril films suggested enantiomeric structures of the hetero-double-helices, derived from (*P*)-pentamer and (*M*)-hexamer (Figure 29 and Figure 30).

**Figure 31:**
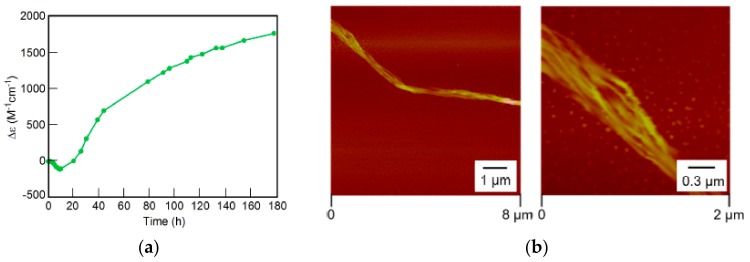
∆ε/time profiles of kinetics in the formation of self-assembly gels by mechanical stirring as shown by (**a**) and AFM images (**b**). Reproduced from reference 101 by permission from John Wiley & Sons.

The process of hetero-double-helix and self-assembly gel formation involved multi-step transitions as indicated by the ∆ε/time profiles (Figure 31). AFM analysis showed sequential formation of fibers, bundles and sheets.

**Figure 32:**
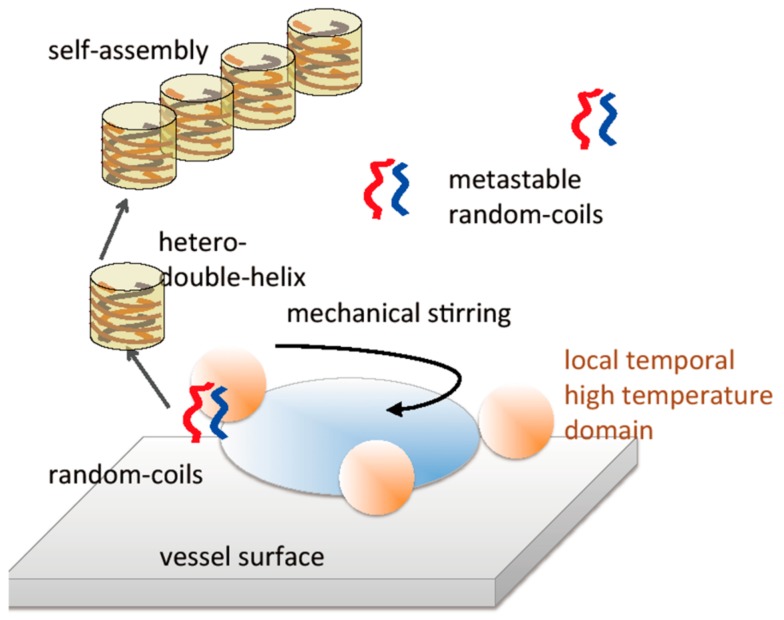
Proposed mechanism of formation of self-assembly gelation by mechanical stirring.

The mechanism of the formation of hetero-double-helices and the self-assembly as a result of mechanical stirring is suggested on the basis of the generation of local and temporal high-temperature domains caused by friction exerted by mechanical stirring (Figure 32). Such heating provides sufficient activation energy for the reaction of metastable random-coils to form hetero-double-helices. The heated domains disappear rapidly because of thermal relaxation and return to the original state and the structural changes occur only in limited domains. Then, the hetero-double-helices self-assemble in the solution phase to form gels.

Solid surfaces are interesting reaction media, where chiral cylindrical molecular complexes exhibit notable structures and dynamics of self-assembly. Metastable homogeneous solutions can be transformed into various heterogeneous liquid-solid materials involving self-assembly on the solid phase depending on conditions.

## 8. Self-Assembly of Nanoparticles with Double-Helix Chiral Cylindrical Molecular Complexes

Along with homo-double-helix formation on flat solid surfaces, the curved solid surfaces of nanoparticles are another interesting reaction medium. Nanoparticles can have much broader surfaces than the flat surfaces and the number of molecules on the surfaces in a dispersed solution is considerably larger, which makes analysis easy [102,103]. The behavior of nanoparticles, such as aggregation and precipitation, is also affected by chiral cylindrical molecular complexes in solution.

### 8.1. Light Emission by Composite Materials of Self-Assembly Gels and Gold Nanoparticles

**Figure 33:**
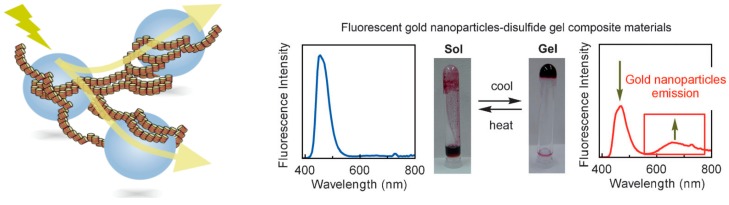
Light emission from composite materials derived from gold nanoparticles and hetero-double-helices self-assembly gels. Changes in visual and fluorescence spectra are shown accompanying the sol-gel transition in response to thermal stimuli. Reproduced from reference 104 by permission from John Wiley & Sons.

Gold nanoparticles with average diameters of 10 nm were reacted with a mixture of the ethynylhelicene (*P*)-trimer and (*M*)-tetramer disulfides (Figure 33), which formed hetero-double-helices on solid surfaces and self-assembly gels in solution [104].

Upon excitation at 365 nm, composite materials produced an emission at 600–800 nm (Figure 33). The emission disappeared upon heating, when the hetero-double-helices and self-assembly gels dissociated; they regenerated upon cooling. Confocal microscopy analysis of dried composite gels exhibited arrays of fluorescent particles at 600 to 700 nm, which indicated that the emission was derived from gold nanoparticles. It is likely that the gel was excited by irradiation and transferred its energy to the nanoparticles, which then emitted light. The self-assembly gels appear to function as antennae for collecting light and transferring energy to arrays of gold nanoparticles, which is interesting because gold nanoparticles are generally considered not to emit light.

### 8.2. Molecular Recognition by Helicene-Grafted Silica Nanoparticles

**Figure 34:**
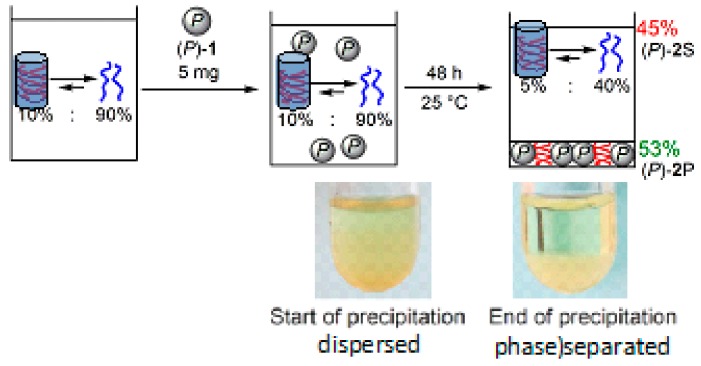
Molecular recognition of homo-double-helices by heliciene-grafted silica nanoparticles, which induces equilibrium shift.

Silica nanoparticles efficiently recognize the structure of the homo-double-helix cylindrical molecular complexes [104,105,106,107,108]. Aminopropylated silica nanoparticles with an average diameter of 70 nm were grafted with (*P*)-helicene acid chloride. The silica (*P*)-nanoparticles recognized the homo-double-helix form of the ethynylhelicene (*P*)-tetramer in solution, adsorbed (*P*)-tetramer and precipitated (Figure 34) [106]. A solution containing 1:9 mixture of homo-double-helices and random-coils of (*P*)-tetramer was treated with (*P*)-nanoparticles for 35–40 h, which formed precipitates. The precipitates contained 53% yield of homo-double-helices and the solution phase contained homo-double-helices and random-coil at 1:9 ratio in 45% yield. The (*P*)-nanoparticles did not precipitate with random-coils but recognized the chiral cylindrical molecular complexes in preference to the irregular random-coils. The removal of the homo-double-helices by precipitation shifted the equilibrium in the solution phase.

**Figure 35:**
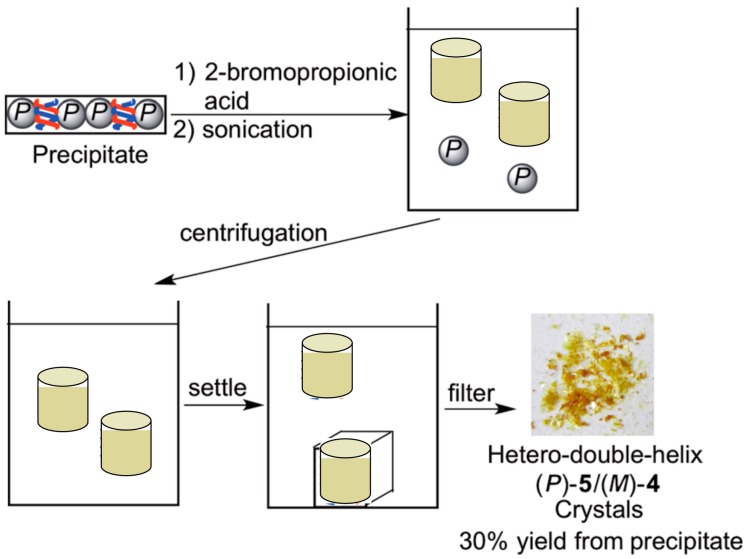
Removal and isolation of hetero-double-helices during self-assembly gelation by silica (*P*)-nanoparticles.

The (*P*)-nanoparticles recognized also hetero-double-helices, which were intermediates in the self-assembly gel formation and removed the chiral cylindrical molecular complexes from the solution phase by precipitation (Figure 35) [107]. A toluene solution of the ethynylhelicene (*P*)-pentamer and (*M*)-tetramer containing (*P*)-nanoparticles was heated to 100 °C and cooled to 25 °C to form a self-assembly gel. Then, (*P*)-nanoparticles, on which the hetero-double-helices were adsorbed in 30% yield, were removed by centrifugation. 

The hetero-double-helices were then isolated by liberation in an appropriate solvent. The (*P*)-nanoparticle precipitates were suspended in 2-bromopropionic acid, sonicated to liberate hetero-double-helices and centrifuged to remove the (*P*)-nanoparticles. When the solution was allowed to settle for 1 h, the hetero-double-helices precipitated in crystalline form. This method could be used to separate intermediates in a chemical reaction.

**Figure 36:**
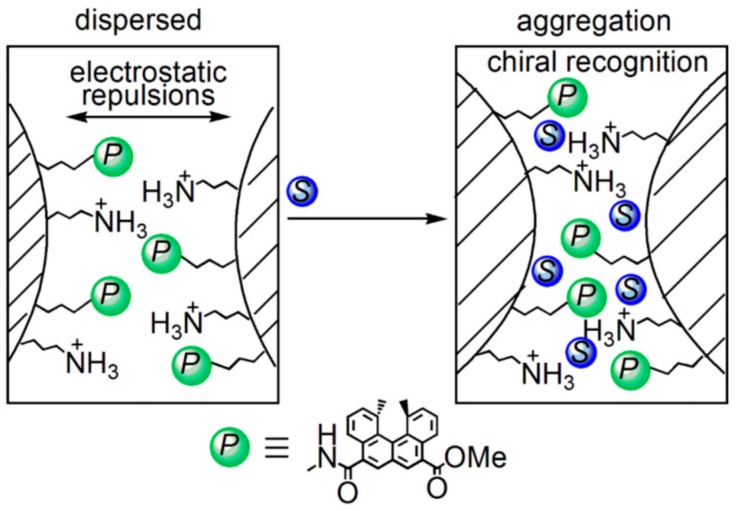
Proposed mechanism of (*P*)-nanoparticles precipitation. S represents chiral cylindrical molecular complex.

A mechanism of (*P*)-nanoparticles precipitation is suggested (Figure 36) [105]. The dispersed state of (*P*)-nanoparticles in solution is derived from the electrostatic repulsions of the protonated aminopropyl groups. Consequently, washing the (*P*)-nanoparticles with triethylamine substantially reduced the stability of the dispersion. The added chiral cylindrical molecular complex is recognized and adsorbed on the solid surface, at which the repulsions between the (*P*)-nanoparticles are reduced, causing them to aggregate and precipitate.

### 8.3. Materials Clocking by Helicene-Grafted Silica Nanoparticles

**Figure 37:**
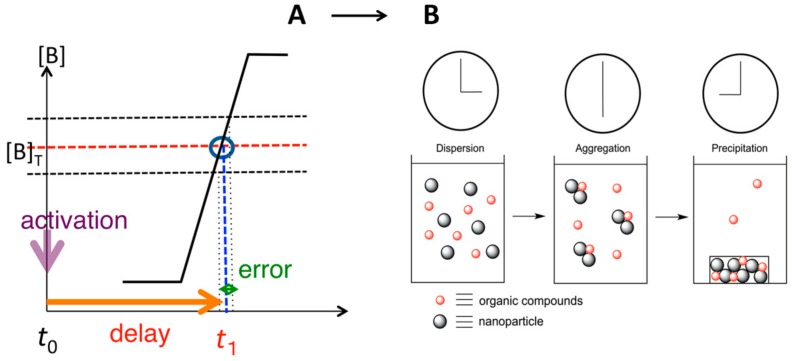
Materials clocking system using precipitation of (*P*)-nanoparticles in the presence of **A**-to-**B** reaction.

Clocking is an important phenomenon in biology, which appears, for example, in the circadian clock [109,110,111,112,113]. It is likely that clocking involves chemical reactions, which occur accurately and precisely at a certain delay time after activation (Figure 37). Usually, chemical reactions are initially fast and later become slow, which is not suitable for clocking, because the error in time will be substantial, especially at later stages. Silica (*P*)-nanoparticles precipitation exhibited stair-shaped kinetics, which can be used for materials clocking [114]. This is a notable kinetic feature in the self-assembly formation of chiral cylindrical molecular complexes.

**Figure 38:**
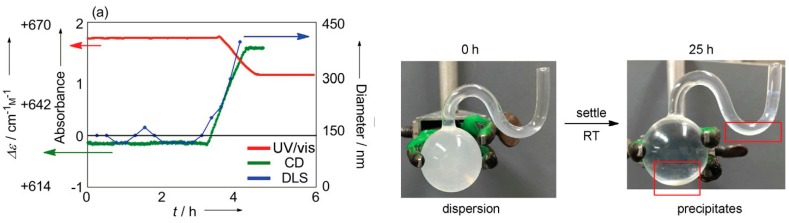
Absorbance/time profiles obtained by UV-vis and CD analyses of mixture of ethynylhelicene (*P*)-pentamer and (*P*)-nanoparticles in trifluoromethylbenzene. Diameter/time profiles (blue line) obtained by DLS analysis. Pictures of the precipitation experiment are also shown. Reproduced from reference 114 by permission from John Wiley & Sons.

A solution of a homo-double-helix (*P*)-pentamer and silica (*P*)-nanoparticles was dispersed by sonication and the metastable dispersion was allowed to settle. Precipitation started from the top part of the solution at 3 h and completed at 4 h (Figure 38). During the initial 3 h, UV-vis absorbance remained unchanged and then an abrupt decrease occurred in a stair-shape manner, which reached a constant value at 4 h. Essentially the same kinetics were observed in the CD, UV-vis and dynamic light scattering (DLS) analysis. The decrease was ascribed to the precipitation of the (*P*)-nanoparticles and adsorption of the (*P*)-pentamer, during which 30% of the (*P*)-pentamer was removed from the solution phase. The experiment was highly reproducible with regard to the start time and the end time of the absorbance change, making it suitable for materials clocking with delayed time.

The homo- and hetero-double-helix cylindrical chiral molecular complexes have a high affinity for the silica nanoparticles surfaces and the materials system exhibits molecular recognition phenomena and materials clocking.

## 9. Homogeneous-Heterogeneous Transitions in Molecular Dispersed Solutions by Self-Catalysis

**Figure 39:**
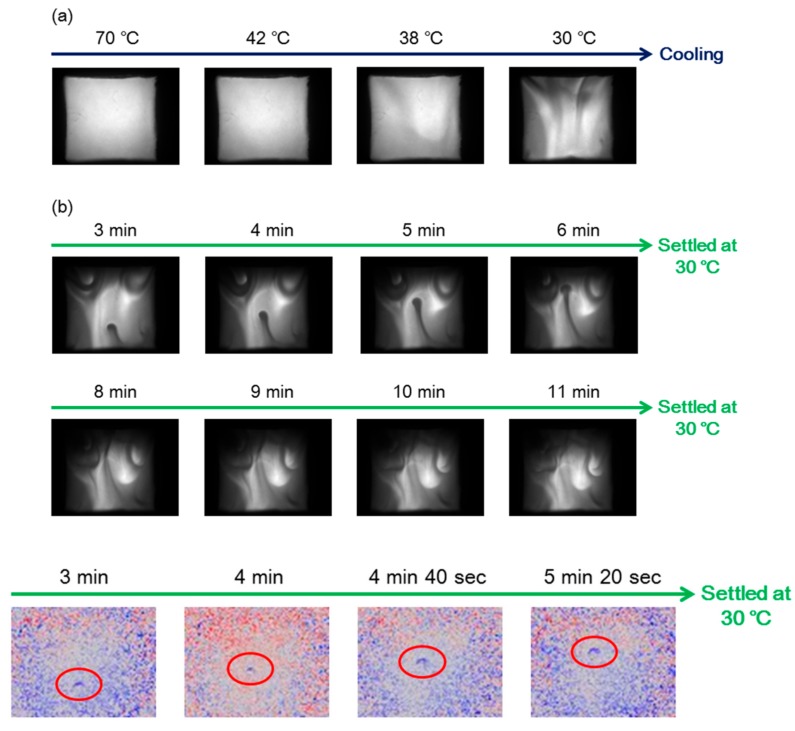
UV-vis images (320 nm) (**a**) and CD images (320 nm) (**b**) of 1:1 mixture of aminomethylenehelicene (*P*)-tetramer and (*M*)-pentamer in fluorobenzene during cooling from 70 to 30 °C. Reproduced from reference 115 by permission from John Wiley & Sons.

The homogeneous-heterogeneous transitions discussed in the previous sections involved the formation of heterogeneous liquid-solid materials. In contrast, the chiral cylindrical molecular complex exhibited spatially homogeneous-heterogeneous transitions in solution by dispersed molecules as indicated by UV-vis/CD imaging, where a self-catalytic network is involved [115]. A metastable homogeneous solution was converted to a metastable heterogeneous state, during which local and temporal patterns were formed by self-catalysis and eventually homogeneous solution at equilibrium formed (Figure 39).

A pseudo-enantiomeric mixture of the aminomethylenehelicene (*P*)-tetramer and (*M*)-pentamer in fluorobenzene was heated to 70 °C. UV-vis imaging analysis showed a homogeneous bright area, consisting of random-coils **A** (Figure 18). Then, the solution was naturally cooled to 30 °C and dark domains 1 mm in size appeared at 38 °C. This observation is consistent with the formation of hetero-double-helices **B**, which exhibits weaker absorption at 320 nm than **A**. At 30 °C because it was a metastable solution, the dark domains moved and rotated changing shape at a rate of approximately 1 mm min^−1^. A meteor like travelling pattern 1 mm in size also appeared from the bottom to the top, moving at a rate of 1 mm min^−1^. The tip of the meteor like travelling pattern was analyzed by CD imaging, revealing a negative ∆ε, which was consistent with **B** formation. Thus, a spatially heterogeneous solution containing **B** was spontaneously formed a homogeneous solution of **A**.

A heterogeneous solution containing **B** was formed by amplification involving a self-catalytic reaction, in which **B** catalyzes the reaction of **A** to become **B**. The result indicated that a self-catalytic chemical reaction at the molecular level can be a spatially heterogeneous phenomenon in macroscopic domains at the millimeter level.

## 10. Conclusions

Chiral cylindrical molecular complexes are an interesting group of organic compounds, which characteristically have anisotropic three-dimensional shapes with large surface areas of the lateral and bottom faces. Chiral cylindrical molecular complexes are obtained from homo- and hetero-double-helices formed by chiral helicene oligomers and the molecular complexes exhibit notable structures and functions in the formation of heterogeneous liquid-solid materials.

Chiral cylindrical molecular complexes exhibit dynamic properties, in that they reversibly associate and dissociate in response to changing conditions such as temperature, concentration, solvent and chemical substances. Notable phenomena appeared with regard to the thermodynamic aspect of their behaviors: reversible transitions between double-helices and random-coils, the predominant formation of hetero-double-helices over homo-double-helices, formation of enantiomeric three-dimensional structures, ordinary and inverse thermo responses, effect of the formation of water cluster and organic solvent clusters, inversion of enantiomeric three-dimensional structures and synthesis of function by multi-domain oligomers. Notable phenomena also appeared with regard to the kinetic aspect of their behavior: sigmoidal and stair-shaped kinetics, solid surface catalysis, chiral symmetry breaking and homogeneous-heterogeneous transitions in molecularly dispersed solutions by self-catalysis.

The chiral cylindrical molecular complexes self-assemble owing to the strong intercomplex interactions, and, as a consequence, provide various heterogeneous liquid-solid materials of gels, vesicles, LLCs, SAM and nanoparticles precipitates, in which solid phases are formed by self-assembly. Notable self-assembly dynamics was observed with regard to the thermodynamic aspect of their behavior, accompanied by molecular complex dissociation and association: self-assembly gelation; 1:1 and 1:2 complex formation providing Type I and II gels, respectively; four-component gel formation by ααββ-etrameric aggregate; light-emitting composite materials consisting of gold nanoparticles and self-assembly gels; molecular recognition, aggregation and precipitation by silica nanoparticles; reversible gel shrinkage due to synthesis of function; and interconversion between ordered structures of self-assembly gels and LLCs. With regard to the kinetic aspect, we observed discontinuous fibril film formation, mechanically induced self-assembly gel formation, materials clocking by silica nanoparticle precipitation and chiral symmetry breaking with self-assembly gel formation.

These functions of heterogeneous liquid-solid materials involving self-assembly materials are programmed into the molecular structures of oligomers and chiral cylindrical molecular complexes. In addition, molecular modification can be employed for the development and tuning of the properties of heterogeneous liquid-solid materials. Such controllable nature of chiral cylindrical molecular complexes is in contrast to that of small molecules, in which small structural changes often substantially affect the properties of heterogeneous liquid-solid materials.

## Figures and Tables

**Table 1:** Two-component gels formation by the pseudo-enantiomeric ethynylhelicene oligomers shown by minimal gelation concentrations (mM). The compounds are shown in terms of the absolute configuration and numbers of helicene. The types of the gels formed by the combinations, type I and II, are also shown by shades in green and blue, respectively.

	(*M*)-1	(*M*)-2	(*M*)-3	(*M*)-4	(*M*)-5	(*M*)-6	(*M*)-7	(*M*)-8
(*P*)-1	C	S	S	S	S	S	Type II	
(*P*)-2		C	S	S	S	S		
(*P*)-3			1	2.5	2.5-5.0	0.25–0.5	1	1
(*P*)-4				1	0.05–0.1	0.05–0.15	1	
(*P*)-5					0.25	0.1	Type I	
(*P*)-6						0.25		

C, crystals; S, solution.
